# Effects of plant natural products on metabolic-associated fatty liver disease and the underlying mechanisms: a narrative review with a focus on the modulation of the gut microbiota

**DOI:** 10.3389/fcimb.2024.1323261

**Published:** 2024-02-20

**Authors:** Tianqi Cai, Xinhua Song, Xiaoxue Xu, Ling Dong, Shufei Liang, Meiling Xin, Yuhong Huang, Linghui Zhu, Tianxing Li, Xueke Wang, Yini Fang, Zhengbao Xu, Chao Wang, Meng Wang, Jingda Li, Yanfei Zheng, Wenlong Sun, Lingru Li

**Affiliations:** ^1^ School of Life Sciences and Medicine, Shandong University of Technology, Zibo, Shandong, China; ^2^ National Institute of Traditional Chinese Medicine Constitution and Preventive Medicine, Beijing University of Chinese Medicine, Beijing, China; ^3^ College of Life Science, Yangtze University, Jingzhou, Hubei, China; ^4^ Institute of Basic Theory for Chinese Medicine, China Academy of Chinese Medical Sciences, Beijing, China; ^5^ The Second Clinical Medical College, Henan University of Chinese Medicine, Zhengzhou, China; ^6^ Basic Medical College, Zhejiang Chinese Medical University, Hangzhou, China

**Keywords:** MAFLD, gut microbiota, plant natural products, metabolite, gut-liver axis

## Abstract

Metabolic-associated fatty liver disease (MAFLD) is a chronic liver disease characterized by the excessive accumulation of fat in hepatocytes. However, due to the complex pathogenesis of MAFLD, there are no officially approved drugs for treatment. Therefore, there is an urgent need to find safe and effective anti-MAFLD drugs. Recently, the relationship between the gut microbiota and MAFLD has been widely recognized, and treating MAFLD by regulating the gut microbiota may be a new therapeutic strategy. Natural products, especially plant natural products, have attracted much attention in the treatment of MAFLD due to their multiple targets and pathways and few side effects. Moreover, the structure and function of the gut microbiota can be influenced by exposure to plant natural products. However, the effects of plant natural products on MAFLD through targeting of the gut microbiota and the underlying mechanisms are poorly understood. Based on the above information and to address the potential therapeutic role of plant natural products in MAFLD, we systematically summarize the effects and mechanisms of action of plant natural products in the prevention and treatment of MAFLD through targeting of the gut microbiota. This narrative review provides feasible ideas for further exploration of safer and more effective natural drugs for the prevention and treatment of MAFLD.

## Graphical Abstract

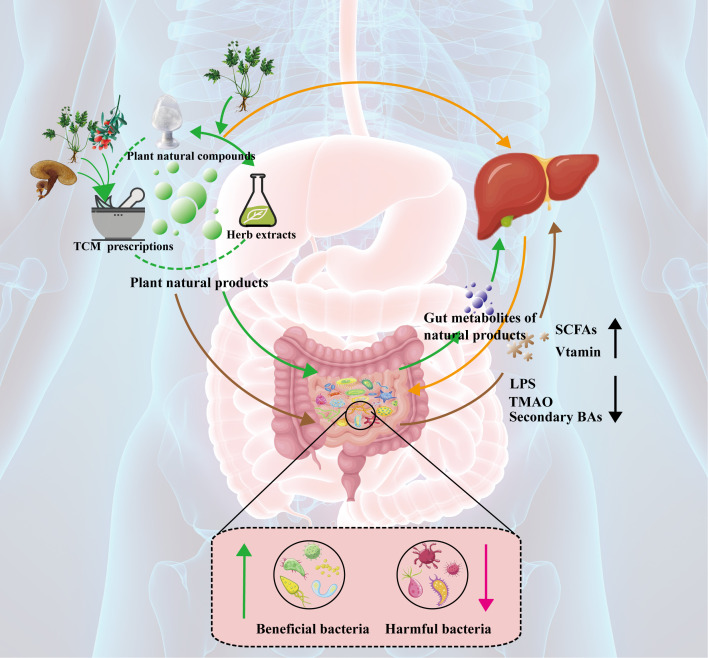

## Highlights

- The gut microbiota may be a new target for MAFLD.
- Plant natural compounds can prevent and treat MAFLD by targeting the gut microbiota.
- Herb extracts could prevent and treat MAFLD by targeting the gut microbiota.
- TCM prescriptions could prevent and treat MAFLD by targeting the gut microbiota.
- Changing the structure and metabolites of the gut microbiota is the mechanism of action of plant natural products.

## 1 Introduction

Metabolic-associated fatty liver disease (MAFLD) is a new term for nonalcoholic fatty liver disease (NAFLD) (Eslam et al., 2020) and is a standard positive diagnosis based on metabolic factors and independent of alcohol use. Currently, the diagnostic criteria for MAFLD are based on evidence of hepatic steatosis (demonstrated by biopsy, imaging or validated serum biomarkers), in addition to one of the following criteria: overweight/obesity, type 2 diabetes mellitus, or metabolic dysregulation defined by the presence of at least two metabolic risk factors, including high waist circumference, hypertension, hypertriglyceridemia, hypo-HDL cholesterolemia, prediabetes, insulin resistance, and elevated high-sensitivity C-reactive protein levels. Recently, the prevalence rate of MAFLD has increased consistently. The global prevalence of MAFLD is already approximately 25% (Younossi et al., 2016), reaching approximately 70% in the obese population (Quek et al., 2022). MAFLD has become the most common chronic liver disease in the world. MAFLD comprises a continuous spectrum of liver diseases, including not simple fatty liver (NAFL) but also dynamic disease that can progress to steatohepatitis (NASH), decompensated cirrhosis and even hepatocellular carcinoma (HCC), and has gradually become an important cause of liver failure and liver transplantation. Moreover, the development of MAFLD often occurs in combination with various metabolic disorders and contributes to the progression of serious diseases, such as gout (Kuo et al., 2010), type 2 diabetes mellitus (T2DM) (Tanase et al., 2020), hypertension (Ma et al., 2021), and cardiovascular disease (Targher et al., 2016). Therefore, it is particularly important to find appropriate and efficient ways to control and treat MAFLD.

Currently, traditional management strategies for MAFLD largely focus on weight reduction and lifestyle modification. Lifestyle interventions consist primarily of dietary interventions and exercise interventions. Most studies of dietary weight loss have shown limited average weight loss (<5%) after 12 months of intervention, and there are some risks associated with long-term ketogenic diet interventions (Anekwe et al., 2020). For exercise interventions, it is recommend that the average adult complete at least 30 min of moderate-intensity aerobic exercise on 1 day and no less than 4 times per week for at least 16 weeks (Shojaee-Moradie et al., 2016). Both interventions (dietary and exercise interventions) are not only long-lasting and slow but also difficult to adhere to, leading to poor patient compliance. Pharmacological interventions are usually used in patients with MAFLD who fail to respond to conventional treatments. Pharmacological interventions prevent the progression of hepatitis and liver fibrosis by reducing liver fat accumulation and alleviating inflammatory damage. Studies have shown that drugs for type 2 diabetes can be used to treat patients with MAFLD (Mitrovic et al., 2022), but there are several toxic side effects. In fact, there are no Food and Drug Administration (FDA)-approved drugs for MAFLD treatment. Therefore, there is an urgent need to find safe and effective anti-MAFLD targets and drugs.

The gut microbiota plays an important role in regulating gut development, regulating host nutrient metabolism, preventing pathogenic bacterial colonization, and maintaining the gut barrier and immune function (Zhang et al., 2015). In recent years, numerous studies have shown that gut ecological dysbiosis is closely associated with the progression of obesity (Fei and Zhao, 2013; Cheng et al., 2022), type 2 diabetes (Ma et al., 2019) and cardiovascular disease (Xu H. et al., 2020). In particular, many studies have indicated that the gut microbiota is associated with the development of MAFLD (Le Roy et al., 2012). There are differences in the gut microbiota between healthy individuals and MAFLD patients, and MAFLD patients have poorer gut microbial ecological diversity and reduced bacterial abundance. Moreover, germ-free mice exhibited significantly reduced sensitivity to diet-induced hepatic steatosis (Bäckhed et al., 2004). Based on these findings, the gut microbiota has become a potential new therapeutic target for MAFLD.

A natural product is defined as “a product derived from a plant, animal or microbial source, also known as natural sources.” (Paola et al., 2023) Normally, plant natural compounds, herb extracts and traditional Chinese medicine (TCM) prescriptions have been considered the main forms of plant natural products. Historically, plant natural products, especially TCMs, have been used since ancient times and in folk medicine for the treatment of many diseases and illnesses. Recently, plant natural products have attracted much attention for the treatment of MAFLD due to their multiple targets and pathways and few side effects. However, their low bioavailability has limited their further development. Many research studies have indicated that only some plant natural products are absorbed in the small intestine, while most of these products reach the colon, where the composition of the gut microbiota can be affected, facilitating the metabolism of the microbiota and alleviating MAFLD. Recent studies have suggested that plant natural products may exert their hypolipidemic and hepatoprotective effects by altering the structure of and metabolite production by the gut microbiota (Zhang N-N. et al., 2023). However, the effects of plant natural products on MAFLD and the underlying mechanisms are also poorly understood and need to be further clarified, especially given the data from the last five years.

Based on the above information and to address the potential therapeutic role of plant natural products in MAFLD, we systematically summarize the effects and mechanisms of action of plant natural compounds, herb extracts and TCM prescriptions in the prevention and treatment of MAFLD through targeting of the gut microbiota. This review summarizes studies from the past 5 years reported in databases, including PubMed, Web of Science, Google Scholar, and X-MOL, which were filtered using the keywords “gut microbiota” and/or “MAFLD”. Those on human-modified substances, such as plant natural product-related nanoparticles or synthetic derivatives, are beyond the scope of this review. Overall, this narrative review provides feasible ideas for the further exploration of safer and more effective natural anti-MAFLD drugs.

## 2 A potential mechanism for the treatment of MAFLD by targeting the gut microbiota and its metabolites

**Figure 1:**
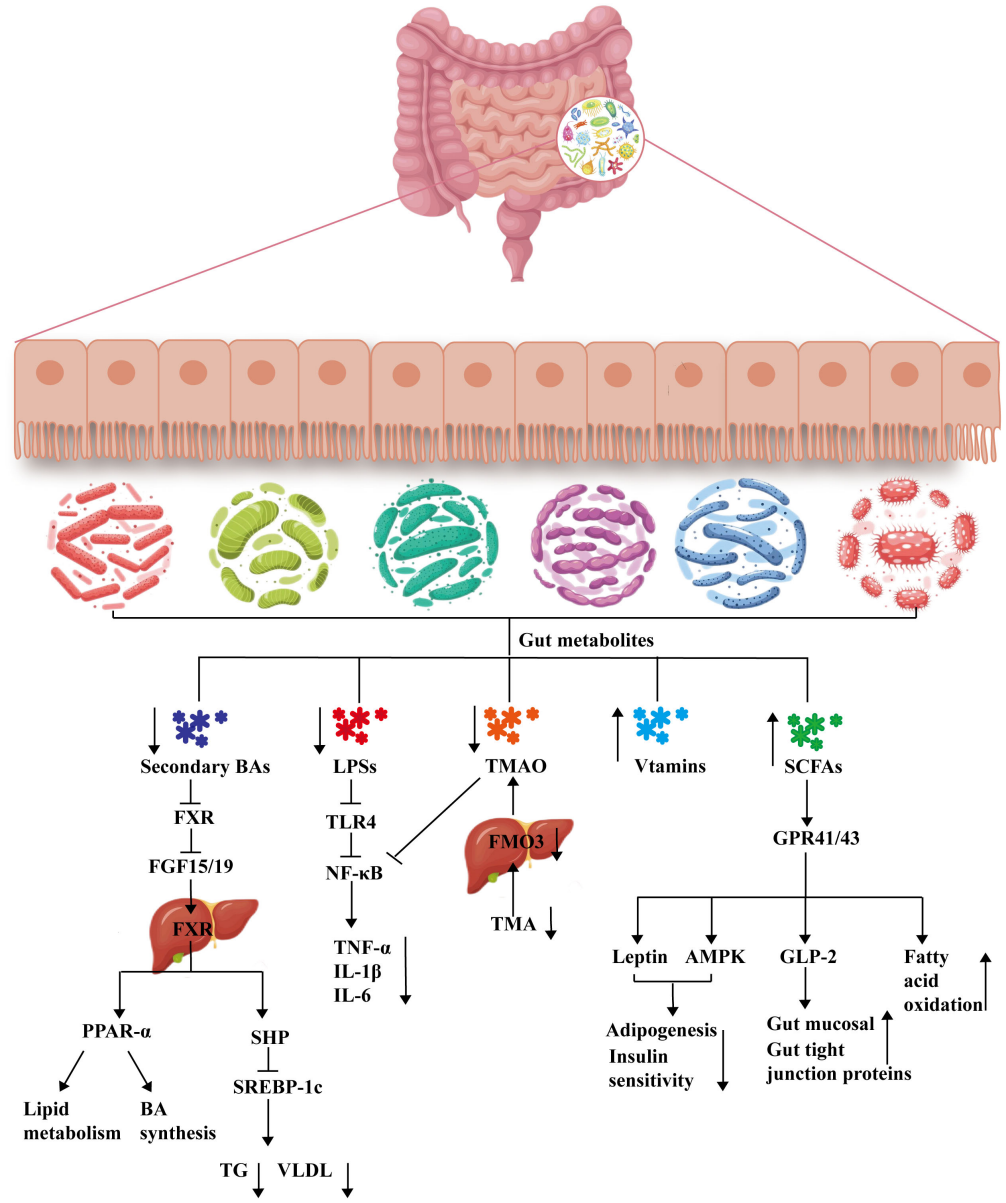
Mechanisms of gut metabolism involved in the treatment of MAFLD. BA, bile acid; FXR, farnesoid X receptor; FGF15/19, fibroblast growth factor 15/19; PPAR-α, peroxisome proliferator-activated receptor alpha; SHP, small heterodimer partner; SREBP-1c, sterol regulatory element binding protein-1c; TG, triglyceride; VLDL, very low-density lipoprotein; LPS, lipopolysaccharide; TLR4, Toll-like receptor-4; NF-κB, nuclear factor kappa-B; TNF-α, tumor necrosis factor alpha; IL-1β, interleukin-1β; IL-6, interleukin-6; TMAO, trimethylamine-N-oxide; TMO, trimethylamine; FMO3, flavin monooxygenase; SCFA, short-chain fatty acid; GPR41/GPR43, mammalian G protein-coupled receptor 41/43; AMPK, adenosine 5’-monophosphate (AMP)-activated protein kinase; GLP-2, glucagon-like peptide-2 receptor.

The gut microbiome is a complex ecosystem composed of bacteria, archaea, fungi, protozoa and viruses that not only participates in nutrient digestion and immune regulation under host physiological and pathological conditions but also serves as a bridge between the gut and other extragut tissues (Kuziel and Rakoff-Nahoum, 2022). Thus, changes in the gut microbiome may affect the health of the host. Many studies have indicated that the gut microbiota is closely associated with the progression of MAFLD (Safari and Gérard, 2019; Hrncir et al., 2021; Shen et al., 2017; Boursier et al., 2016). For example, the ratio of Firmicutes to Bacteroides is greater in patients with MAFLD than in healthy people, and the abundances of Proteobacteria and Enterobacteriaceae increase while that of Ruminococcaceae decreases in these patients (Boursier et al., 2016). The abundance of *Prevotella* decreases significantly with increasing liver inflammation (Chen and Vitetta, 2020). However, the mechanism through which the gut microbiota affects MAFLD remains incompletely understood. With the development of metabolomics technology, changes in gut metabolites during the development of MAFLD have gradually been revealed (Chen and Vitetta, 2020). Among them, the roles of bile acid (BAs), lipopolysaccharides (LPSs), short-chain fatty acids (SCFAs), trimethylamine-N-oxide (TMAO) and vitamins have received widespread attention. These findings may lead to the identification of potential mechanisms of action for the treatment of MAFLD and to new feasible ideas for intervention therapy for MAFLD, as shown in **Figure 1**.

### 2.1 The gut microbiota may ameliorate MAFLD by regulating the BA pool composition

Bile acids (BAs) are cholesterol metabolites that play an important role in the balance of cholesterol and energy metabolism and the absorption of nutrients in the small intestine. The liver is the main site for BA synthesis. BAs are synthesized in the liver and subsequently secreted into the bile duct and stored in the gallbladder. After eating, the gallbladder contracts, and the stored BAs are expelled into the small intestine. In the small intestine, 95% of the BAs are reabsorbed by the small intestine and returned to the liver via the hepatic portal vein, with another approximately 5% exiting the body in the feces or entering systemic circulation (Di Ciaula et al., 2017). BAs act as emulsifiers to promote the absorption and transport of lipids and other substances in the small intestine and as indispensable signaling molecules that bind to a variety of receptors, including the nuclear receptor farnesoid X receptor (FXR), vitamin D receptor (VDR), pregnane X receptor (PXR) and the membrane-bound G protein-coupled receptor Takeda G protein-coupled receptor 5 (TGR5), which exert essential effects to regulate the balance of BA metabolism, glycolipid metabolism and energy metabolism (Perino and Schoonjans, 2022). Recently, much attention has been given to the interaction between BAs and the gut microbiota (Long et al., 2017). Gut microbes can directly modify BAs, such as bacteria that produce bile salt hydrolases (BSHs), which catalyze the conversion of primary bile acids to secondary bile acids, resulting in decreased levels of coupled BAs that may activate intestinal FXR expression and promote hepatic steatosis (Yang and Wu, 2022). Most studies have suggested that BA levels are elevated and that the BA composition is substantially altered in the serum and liver of MAFLD patients (Ferslew et al., 2015; Kalhan et al., 2010). It has also been established in animals that changes in the expression of BA-metabolizing enzymes and transporters that occur with the progression of NASH-related liver fibrosis lead to an increase in the plasma total bile acid (TBA) concentration (Suga et al., 2019). Based on the above information, the gut BA pathway might participate in the treatment of MAFLD.

Changes in composition are the main way that the gut microbiota alters BAs, and these changes affect the efficiency of binding of BAs to their receptors. For example, the order in which BAs bind and activate FXR is chenodeoxycholic acid > lithocholic acid > deoxycholic acid > cholic acid; the most efficacious BA ligands for TGR5 are in the order lithocholic acid > deoxycholic acid > chenodeoxycholic acid > cholic acid (Chiang, 2013). BAs regulate lipid and glucose metabolism mainly through the receptors FXR and TGR5 (Schaap et al., 2013). On the one hand, BAs can reduce triglyceride levels through the pathways of the FXR and the small heterodimer partner (SHP), as well as sterol regulatory element-binding protein 1c (SREBP-1c) (Watanabe et al., 2004). In addition, FXR activation promotes the expression of peroxisome proliferator-activated receptor alpha (PPAR-α), which facilitates the regulation of lipid metabolism, glucose homeostasis and anti-inflammatory activity (Carr and Reid, 2015; Pineda Torra et al., 2003). On the other hand, BAs not only activate TGR5 to improve insulin sensitivity and reduce obesity (Brighton et al., 2015; Thomas et al., 2009) but also inhibit the expression of cytochrome P450 7A1 (CYP7A1) by activating FXR and TGR5, thus inhibiting BA synthesis (Goodwin et al., 2000). Many studies have shown that TGR5 or FXR agonists can reduce lipogenesis, alleviate cholesterolemia, induce energy expenditure, and reduce liver inflammation (Pineda Torra et al., 2003; McMahan et al., 2013; Jadhav et al., 2018). BAs affect not only the gut-liver axis but also the gut-brain axis. BA-TGR5 signaling reduces the expression of Agouti-related protein (AgRP)/neuropeptide Y (NPY) and temporarily blocks the release of neuropeptides in AgRP/NPY neurons, which in turn inhibits feeding behavior, which may also be relevant to the treatment of MAFLD (Perino et al., 2021). Therefore, activation of FXR or TGR5 by regulating the gut microbiota to change the BA pool composition is expected to be the main therapeutic mechanism for the treatment of MAFLD.

### 2.2 Increased concentrations of SCFAs may ameliorate MAFLD in the gut microbiota

Under the action of anaerobic microorganisms in the mammalian colon, carbohydrates are degraded and fermented to produce large amounts of short-chain fatty acids (SCFAs), and acetic acid, propionic acid, and butyric acid make up approximately 90%-95% of the total SCFAs. SCFAs play an active role in the pathogenesis of MAFLD through portal vein entry into the liver and regulation of the inflammatory response, lipid metabolism and glucose metabolism (Zhou and Fan, 2019). SCFAs can also protect the gut barrier (Liu et al., 2020). Studies have shown that MAFLD patients have fewer SCFA-producing bacteria in the gut and decreased levels of SCFAs in the feces (Raman et al., 2013). In addition, numerous studies have demonstrated that SCFA or butyrate supplementation can repair the gut barrier and ameliorate NASH (Jin et al., 2015; Zhou et al., 2017; Gart et al., 2021; Ye et al., 2018). Therefore, the gut SCFA pathway might be a potential target for the treatment of MAFLD.

The specific receptors for SCFAs identified are mainly mammalian G protein-coupled receptor 41 (GPR41), mammalian G protein-coupled receptor 43 (GPR43), and G protein-coupled receptor 109 A (GPR109A). GPR41 and GPR43 are highly expressed in adipocytes, enteroendocrine cells and immune cells (polymorphonuclear cells and macrophages) (Maslowski et al., 2009), while GPR109A is expressed in adipocytes, hepatocytes and colon cells (Singh et al., 2014). These receptors usually mediate anti-inflammatory effects directly (Thorburn et al., 2014). Recent studies have shown that mammalian G protein-coupled receptors, especially GPR41, GPR43, and GPR109A, play important roles in metabolism, inflammation, and disease regulation and may be potential new drug targets for the treatment of certain metabolic diseases (Tan et al., 2014; Sekiguchi et al., 2015). The different SCFAs had the following order of activity against GPR43: propionic acid (C3) ≥ acetic acid (C2) = butyric acid (C4) > valeric acid (C5) > capric acid (C6) = formic acid (C1) (Brown et al., 2003; Nilsson et al., 2003). Activation of GPR43 directly inhibits lipid degradation (Hong et al., 2005; Ge et al., 2008). Thus, the gut microbiota may regulate lipid metabolism by affecting the SCFA composition and the expression of receptors in the gut. Moreover, SCFAs may inhibit adipogenesis through GPR41 or GPR43 by stimulating leptin secretion from white adipocytes in mice and suppressing appetite (Xiong et al., 2004; Zaibi et al., 2010). On the other hand, SCFAs also ameliorate gut inflammation and reduce gut mucosal injury. SCFAs may promote gut mucosal growth and development by inducing human glucagon-like peptide-2 activation; SCFAs may inhibit gut inflammation by activating GPR43 to protect the liver from portal vein-derived gut microbes while decreasing insulin sensitivity in adipose tissue and activating the hepatic adenosine 5’-monophosphate (AMP)-activated protein kinase (AMPK) signaling pathway, which directly or indirectly plays a protective role in liver health (Zhu et al., 2014). In conclusion, SCFAs and their receptors, which are regulated by the gut microbiome, play essential roles in regulating lipid metabolism and the inflammatory response, thereby improving the pathological progression of MAFLD.

### 2.3 The gut microbiota may ameliorate MAFLD by reducing LPS levels

LPSs are glycolipids that can be found on the surface of bacteria and are produced mainly by gram-negative bacteria. LPSs are endotoxins that are transported through the serum circulation to target tissues and are recognized by immune cells. Most LPSs activate inflammatory signaling pathways to secrete proinflammatory factors, cause the body to enter a state of chronic low-grade inflammation, induce metabolic abnormalities, and exert few anti-inflammatory effects through immune cells (Bertani and Ruiz, 2018). The gut microbiota is the main source of LPSs in healthy individuals (Candelli et al., 2021). Differences in the composition of the gut microbiota determine whether LPSs are toxic. For example, LPS produced by *Bacteroides* is harmless (Poli and Orange, 2017), while LPS produced by *Escherichia coli* is highly toxic and increases the level of fecal calprotectin, which is a marker of gut inflammation (Orivuori et al., 2015). Moreover, the serum LPS concentration was found to be positively correlated with the abundance of the aerobic bacteria *Escherichia coli* and *Enterococcus* and negatively correlated with the abundance of the anaerobic bacteria *Lactobacillus*, *Bifidobacterium* and *Bacteroides*, which is consistent with the results of microbiota changes in MAFLD patients (Xue et al., 2017).

Toll-like receptor 4 (TLR4) is an important member of the TLR family. Studies have shown that TLR4 is a key pattern recognition receptor for LPSs and plays an important role in the connection between the innate immune system and metabolic syndrome (Csak et al., 2011; Maddie et al., 2022; Sharifnia et al., 2015). During the progression of MAFLD, serum LPS levels and hepatic TLR4 expression were elevated in patients, while the gut microbiota diversity and biological colonization resistance of gut microorganisms were decreased (Thuy et al., 2008; Dapito et al., 2012). In addition, the correlation between hepatic TLR4 and the gut microbiota also showed the above trend. These findings suggest that LPSs and TLR4 are key molecules in the pathogenesis of MAFLD and that liver injury mediated by the LPS-TLR4 signaling pathway may be involved in the progression of MAFLD. Many studies have indicated that gut LPS and TLR4 activation is associated with diet-induced MAFLD onset, and the use of LPSs can serve as noninvasive tools for the diagnosis and grading of MAFLD severity in overweight and obese patients (Hegazy et al., 2020). Therefore, activation of TLR4 by regulating the gut microbiota to reduce LPS levels could regulate lipid metabolism and the inflammatory response and is expected to be the main therapeutic mechanism for the treatment of MAFLD.

### 2.4 The gut microbiota may affect MAFLD by regulating TMAO

Trimethylamine-N-oxide (TMAO) is an important product of the enterohepatic axis that is produced by the metabolism of trimethylamine (TMA) by the gut microbiota and enters the liver via the enterohepatic axis, where it is then oxidized by enzymes such as flavin-containing monooxygenase 3. Clinical studies have shown a significant increase in the serum TMA, TMAO and choline levels in patients with MAFLD compared to those in healthy individuals and that there is an association between high circulating TMAO concentrations and MAFLD as well as NASH (Theofilis et al., 2022; Flores-Guerrero et al., 2021; León-Mimila et al., 2020; Shi et al., 2022). TMAO may promote the progression of MAFLD through several mechanisms. For example, TMAO may directly enhance the development of MAFLD by affecting oxidative stress (Li X. et al., 2021); TMAO stimulation leads to increased expression of unfolded protein response-related proteins (glucose-regulated protein 78, X-box binding protein 1, and Derlin-1) (Shi et al., 2022), which may lead to hepatocyte lipid metabolism disorders and inflammation, causing the development and progression of MAFLD and ultimately to death (Lebeaupin et al., 2018; Song and Malhi, 2019). Furthermore, serum TMAO levels were found to be positively associated with total serum BA levels and hepatic CYP7A1 expression, suggesting that TMAO can increase BA synthesis and shift hepatic BA components toward FXR antagonistic activity, thereby exacerbating hepatic steatosis (Tan et al., 2019). However, it has also been shown that TMAO supplementation enhances the ability to repair tissue damage by increasing the number of endothelial cells in mice, thereby enhancing the integrity of the blood–brain barrier and protecting it from inflammatory damage, which may be beneficial in ameliorating NASH. For example, Zhou et al. reported that TMAO supplementation restored gut microbiota diversity, reduced liver fibrosis, and protected vascular function in mice, suggesting a possible protective effect of TMAO metabolic retrotransposition in the gut (Zhou et al., 2022). Therefore, reducing elevated TMAO levels by regulating the gut microbiota is expected to be the main therapeutic mechanism for the treatment of MAFLD.

### 2.5 The gut microbiota may affect MAFLD by regulating gut vitamins

Vitamins are essential nutrients for the normal growth of humans and animals, and deficiency and overdose can lead to metabolic disorders and reduced growth performance (Brennan, 2006). The human body cannot synthesize most vitamins and must obtain them from the diet or rely on symbiotic bacteria in the gastrointestinal tract for their synthesis (Hill, 1997). For example, *Lactobacillus* and *Bifidobacterium* species are capable of synthesizing most water-soluble B vitamins, and *E. coli* is capable of synthesizing vitamin K. The development of MAFLD has been shown to disrupt the gut microbiota; therefore, MAFLD may affect the synthesis of vitamins in the body through the gut microbiota. Clinical studies have indicated that vitamin D (VD) levels are markedly lower in patients with MAFLD than in those without this disease (Chung et al., 2016; Tian et al., 2020). The biological roles of VD include not only maintaining the balance of calcium and phosphorus metabolism in the body and participating in bone reconstruction but also reducing insulin resistance, modulating immunity, protecting cardiovascular health, and exerting antifibrosis and anti-inflammation effects (Chun et al., 2019; Whiting and Calvo, 2021). Numerous epidemiological investigations have shown that VD deficiency is closely associated with MAFLD, cirrhosis and obesity, and there is also evidence that VD has a protective effect against MAFLD (Tian et al., 2020; Du et al., 2023; Barchetta et al., 2011; Ciardullo et al., 2023; Barchetta et al., 2020; Barchetta et al., 2017; Pop et al., 2022). Furthermore, VD supplementation can alter the abundance and diversity of the gut microbiota. Zhang et al. reported that VD supplementation could mitigate MAFLD by increasing the relative abundances of *Prevotella* and Porphyromonadaceae and decreasing the relative abundances of *Mucispirillum*, *Acetatifactor*, *Desulfovibrio* and *Oscillospira* (Zhang X-L. et al., 2023). In addition to VD, numerous studies have shown that supplementation with vitamin B12 (Li L. et al., 2022), vitamin C (Lee et al., 2022), vitamin E (Perumpail et al., 2018), or a vitamin-rich diet could alleviate the progression of MAFLD. Therefore, regulating the gut microbiota by vitamin supplementation may be an additional modality for MAFLD treatment.

### 2.6 Other metabolites

In addition to the above metabolites, the gut microbiota secretes metabolites such as sphingolipids, amino acids, phenolic acids, and ethanol. However, the relationship between these metabolites and the progression of MAFLD requires further investigation. In summary, alterations in the gut microbiota and its metabolites may be potential therapeutic targets for MAFLD.

## 3 Plant natural products ameliorate MAFLD by regulating the gut microbiota

Plant natural products, including plant natural compounds, herb extracts and TCM prescriptions, have attracted much attention in the treatment of MAFLD due to their effects on multiple pathways and multiple targets and their few adverse reactions. Plant natural products can not only affect the gut microbial composition but also regulate gut microbial metabolism. This review systematically summarizes the effects and mechanisms of action of plant natural compounds, herb extracts and TCM prescriptions in the prevention and treatment of MAFLD through the targeting of the gut microbiota reported in studies published from 2017 to 2023 (**Table 1**).

**Table 1:** Mechanism of action of plant natural compounds and herb extracts in the treatment of MAFLD involves modulating the gut microbiota and its metabolites.

Category	Compound name	Experimental subject	Experimental design	Modulation target (gut microbiota structure)		Modulation target	Ref.
Flavones	Baicalin	Male C57BL/6 J mice	Fed with HFD; at the same time, baicalin, 200 mg·kg^-1^·d^-1^ was administered for 15 weeks	*Lactobacilli*, Butyric acid-producing bacteria	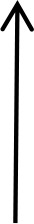	BA metabolism; FXR expression; TGR5 expression	(Liu et al., 2022; Ju et al., 2019; Hu et al., 2021)
	Luteolin	Male Wistar rats	HFD fed with luteolin, 25,50, or 100 mg·kg^-1^ ·d^-1^ for 8 weeks; HFD was fed for 12 weeks, and luteolin was administered continuously from the fifth week	Desulfovibrionacea, Coriobacteriaceae; *Lactobacillus*, *Bifidobacterium*	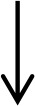 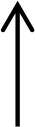	LPS production; SCFA production; Gut barrier; TLR4/NF-κB signaling pathway; LXR-SREBP-1c signaling pathway	(Liu X. et al., 2021)
	Hyperoside	Male Wistar rats	HFD fed concurrently with hyperoside, 0.6 or 1.5 mg·kg^-1^·d^-1^ for 20 days	Gut microbiota involved in BSH activity	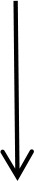	Hepatic FXR; BA metabolism and excretion; Cholesterol metabolism	(Wang S. et al., 2021)
	Myricetin	Male Wistar rats	Fed HFD containing 0.5% myricetin for 12 weeks; antibiotic treatment for one week, subsequently FMT with myricetin for 2 weeks and fed HFD for 12 weeks	SCFA-producing bacteria *Allobaculum*; Butyric acid-producing bacteria	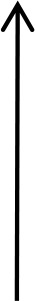	LPS/TLR4/NF-kB pathway; Gut barrier; Lipid synthesis	(Sun et al., 2021)
	Quercetin	Male C57BLKS/J background db/db and db/m mice	db/db mice were fed with a normal diet for 8 weeks as the model group; db/db mice were administered 100 mg·kg^-1^·d^-1^ quercetin for 8 weeks	Increased beneficial bacteria, inhibited harmful bacteria, reduced the ratio of Firmicutes to Bacteroidetes		BA and cholesterol metabolism; FXR/TGR5 signaling pathway; LPS/TLR4 signaling pathway; TMAO metabolism	(Wang T. et al., 2023; Yang et al., 2019; Chen et al., 2021; Porras et al., 2016)
	Dihydromyricetin	Male C57BL/6 mice	HFD supplemented with dihydromyricetin, 50, 150, or 450 mg·kg^-1^·d^-1^ for 16 weeks	*Alistipes*, *Mucispirillum*, *Oscillibacter*	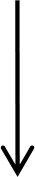	Metabolic endotoxin; BA metabolism; Gut barrier; FXR-related signaling pathway	(Tong, 2018)
	Silybin	Male C57BL/6J mice	HFD fed with silybin, 100,300 mg·kg^-1^ ·d^-1^ for 8 weeks; HFD feeding was performed for 17 weeks, and silybin was administered from the tenth week	SCFA-producing *Blautia*, *Bacteroides*, *Akkermansia*; *Alloprevotella*, *Lactobacillus*	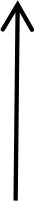 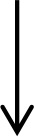	Gut barrier; Mitochondrial function; Apoptosis and oxidative stress	(Li X. et al., 2020)
	Tectorigenin	Male C57BL/6 N mice	HFD fed concurrently with tectorigenin, 25, 50 mg·kg^-1^·d^-1^ for 6 weeks	*Akkermansia*; *Turicibacter*, *Dubosiella*, *Faecalibaculum*	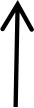 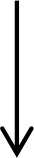	BA metabolism; LPS production; Gut barrier;LPS/TLR-4/NF-κB/TNF-α pathway; Gut FXR	(Duan et al., 2022)
	Puerarin	Male C57BL/6 mice	Fed with MCD; at the same time, puerarin, 0.2 g·kg^-1^·d^-1^ was administered for 4 weeks	Butyrate producing bacteria *Roseburia*; *Akkermansia muciniphila*; LPS producing bacteria *Helicobacter*	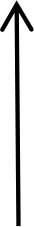 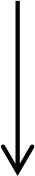	Gut barrier; Liver oxidation; Pyrimidine metabolism; One-carbon metabolism; Amino acid metabolism;Glycolysis, tricarboxylic acid cycle; Synthesis and degradation of ketone bodies	(Gong et al., 2021; Wang L. et al., 2019)
	Nobiletin	Male C57BL/6 mice	Fed with HFHS; at the same time, nobiletin, 100 mg·kg-1·d-1 was administered for 12 weeks	*Allobaculum stercoricanis*, *Lactobacillus casei*	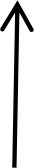	Myristoleic acid metabolism; Primary bile acid biosynthesis and PPAR signaling pathways	(Li et al., 2023)
	Naringin	Male C57BL/6 mice	Fed with HFD; at the same time, 0.07% naringin was administered for 8 weeks	*Allobaculum*, *Alloprevotella*, *Butyricicoccus*, *Parasutterella*, *Lachnospiraceae NK4A136 group*; Campylobacter, Coriobacteriaceae UCG-002, Faecalibaculum, Fusobacterium	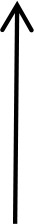 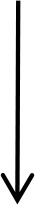	Lipid synthesis; LPS production	(Mu et al., 2020)
Nonflavonoid phenols	Curcumin	Male C57BL/6 mice	HFD supplemented with curcumin, 125 mg·kg^-1^·d^-1^ for 10 weeks	*Desulfovibrio*; *Akkermansia*, SCFA-producing bacteria *Bacteroides*, *Parabacteroides*, *Alistipes*, *Alloprevotella*	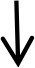 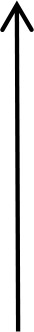	BA metabolism; Nrf2/FXR/LXR-α signaling pathway; SCFA production;AMPK-mTOR signaling pathway; Gut barrier integrity	(Li S. et al., 2021; Yan et al., 2018; Li R. et al., 2020)
	Resveratrol	Male C57BL/6 J mice	HFD with resveratrol, 300 mg·kg^-1^·d^-1^ for 16 weeks	*Desulfovibrio*, *Lachnospiraceae NK4A316 group*, *Alistipes*; SCFA-producing bacteria *Allobaculum*, *Blautia*	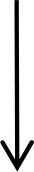 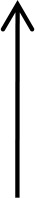	Gut barrier; Gut metabolites 4-hydroxyphenylacetic acid and 3-hydroxyphenylpropionic acid	(Wang et al., 2020a; Wang P. et al., 2019; Wang et al., 2020b; Yin et al., 2020)
Lignin	Schisantherin A	Male C57BL/6J mice	Fed with HFD; at the same time, schisantherin A was administered for 6 weeks	Firmicutes; Bacteroidetes	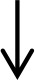 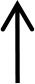	Gut barrier; Gut inflammation; LPS production;LPS-TLR4 signaling pathway	(Yu S. et al., 2022)
Alkaloids	Berberine	WT and FXR^int-/-^ mice with a C57BL/6J background Male C57BL/6J mice and FXR^-/-^mice on a C57BL/6J background	HFD with berberine, 150 mg·kg^-1^ ·d^-1^ for 8 weeks; FXR^int-/-^ mice were fed with HFD and subsequently with berberine, 150 mg·kg^-1^ ·d^-1^ for 8 weeks HFD-DSS feeding with berberine, 150 mg·kg^-1^ ·d^-1^ for 4 weeks, HFD-DSS feeding for 16 weeks, berberine was administered from the thirteenth week	BSH-producing bacteria *Clostridiales*, Lactobacillaceae, *Bacteroidales*	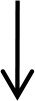 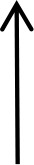	Gut FXR pathway; CD36 expression; BA metabolism; Lipid synthesis and metabolism; BSH activity Gut FXR/FGF15/NF-κB pathway; BA metabolism	(Sun et al., 2016) (Shu et al., 2021)
	Nuciferine	Male Sprague–Dawley rats	HFD feeding with nuciferine, 10 or 25 mg·kg^-1^ ·d^-1^ for 8 weeks	Mucus-associated microbes *Akkermansia muciniphila*, Ruminococcaceae; SCFA-producing bacteria; LPS-producing microbiota Desulfovibrionacea	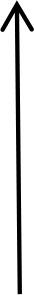 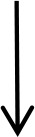	Ileal and liver FXR signaling pathway; BA metabolism; Gut barrier; SCFA production; LPS/TLR4/MyD88/NF-κB signaling pathway	(Sun et al., 2022; Fan et al., 2022; Wang Y. et al., 2020)
	Capsaicin	Male Sprague–Dawley rats	Fed with HFD; at the same time, 0.05% and 0.1% capsaicin were administered for 4 weeks	*Bacteroidales S24-7*, *Akkermansia*, *Allobaculum*; *`Desulfovibrio*, *Lactobacillus*	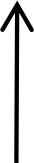 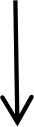	BA metabolism; SCFA production; Liver FXR/FGF15 signaling pathway; Inflammatory signaling pathway;Lipid synthesis and metabolism	(Gong et al., 2022)
	Betaine	Female Kunming mice	HFD and 1% betaine for 23 weeks	*Akkermansia muciniphila*, *Lactobacillus*, *Bifidobacterium*	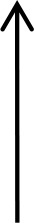	SCFA production; TLR4/MyD88 signaling pathway; TMAO metabolism; Gut barrier	(Du et al., 2021; Wang F. et al., 2019)
	Sinapine	Male C57BL/6J (B6) mice	HFD fed concurrently with sinapine (500 mg·kg^-1^·d^-1^ rapeseed oil, ≥98% purity) in rapeseed oil for 12 weeks	Lactobacillaceae, Akkermansiaceae, *Blautia*; Desulfovibrio	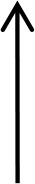 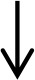	SCFA production; Endotoxin production; SCFA-GPR43 inflammatory pathways	(Li Y. et al., 2019)
Saponins	Astragaloside IV	Male C57BL/6 N mice	HFD with 12.5, 25, or 50 mg·kg^-1^ ·d^-1^ astragaloside IV for 12 weeks	BSH-expressing bacteria	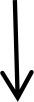	BSH activity; Liver FXR/SHP signaling pathway; Ileal FXR/FGF15 signaling pathway; BA metabolism	(Zhai et al., 2022)
	Ilexsaponin A_1_	Male C57BL/6 mice	HFD with ilexsaponin A_1,_ 120 mg·kg^-1^·d^-1^ for 8 weeks	BSH-expressing bacteria	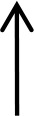	BSH activity; Gut FXR pathway; BA metabolism; LPS production; Inflammatory signaling pathway	(Zhao et al., 2021)
	Ganoderic acid A	Male specific pathogen-free Kunming mice	HFD with ganoderic acid A, 15 or 75 mg·kg^-1^·d^-1^ for 8 weeks	*Eisenbergiella tayi*, *Alistipes, senegalensis*, *Oscillibacter valericigenes*, *Bacteroides acidifaciens*, *Mucispirillum schaedleri*, *Bacteroides eggerthii*; *Parabacteroides goldesteinii*, *Anaerotruncus colihominis*, *Barnesiella intestinihominis*, *Lactobacillus murinus*	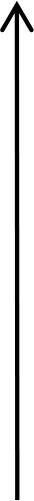 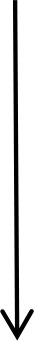	BA synthesis and excretion; SCFA production; Lipid oxidation and liver inflammation; Liver FXR expression	(Guo et al., 2020)
	Ursolic acid	Male hamsters	HCD with 0.2% or 0.4% ursolic acid for 6 weeks	SCFA-producing bacteria *Bifidobacterium*	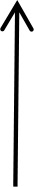	LPS production; SCFA production; Cholesterol metabolism	(Hao et al., 2020)
	Soybean saponin A_2_	Male C57BL/6J (B6) mice	MCD with soybean saponin A_2_ 1, 50 or 100 mg·kg^-1^·d^-1^ for 16 weeks	Erysipelotrichaceae, *Faecalibaculum*; *Desulfovibrionaceae (Desulfovibrio)*	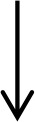 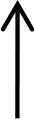	BSH activity; BA metabolism; Ileum FXR/FGF15 signaling; Gut inflammation;“gut-liver axis”	(Xiong et al., 2021)
Polysaccharides	α-D-1,3-glucan	Male C57BL/6J mice	Fed HFD supplemented with LPS at 200 μg/kg per day in autoclaved water (LPS was added for 4 weeks and stopped for 2 weeks) from week 4 to week 16; at the same time, 50 or 100 mg·kg-1·d-1α-D-1,3-glucan was administered for 16 weeks	*Lactobacillus*, *Phocea*, *Ruthenibacterium*, *Flavonifractor*, *Oscillabacter*, *Flintibacter*, *Butyricicoccus*	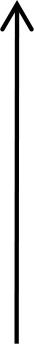	SCFA production; LPS production; Gut barrier; Gut inflammation	(Li Q. et al., 2022)
	MDG-1	Male C57BL/6J mice	HFD with 2‰, 4‰, or 8‰ MDG-1 for 8 weeks, HFD was fed for 16 weeks, and MDG-1 was administered from the ninth week	SCFA-producing bacteria; *Akkermansia muciniphila*; Endotoxin-producing pathogens	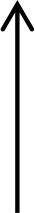 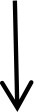	Endotoxin metabolism; SCFA production; Inflammatory response; Liver lipid metabolism	(Wang X. et al., 2019; Zhang L. et al., 2021)
Plant extracts	Gypenosides	Male Sprague–Dawley rats Male C57BL/6 specific pathogen-free (SPF) mice	HFD with gypenosides. 50,100, or 150 mg·kg^-1^ ·d^-1^ for 8 weeks HFD with gypenosides, 100 mg·kg^-1^ ·d^-1^ for 4 weeks; HFD was fed for 14 weeks, and gypenosides were administered from the eleventh week	SCFA-producing bacteria *Akkermansia*, *Bacteroides*, *Parabacteroides*; *Desulfovibrio*, *Escherichia-Shigella*, *Helicobacter*	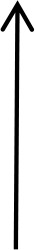 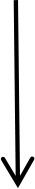	SCFA production; LPS production; Gut inflammation BA metabolism; Liver FXR/SHP signaling; TLR4/NF-κB signaling; TMAO metabolism	(Zhong F-W. et al., 2022) (Li H. et al., 2020)
	*Panax notoginseng* total saponins	Male C57BL/6J mice	ob/ob and HFD-fed mice were fed for 4 weeks; *Panax notoginseng* total saponins at 800 mg·kg^-1^·d^-1^ were administered from 5 weeks to 12 weeks	*Akkermansia muciniphila*, *Parabacteroides distasonis*	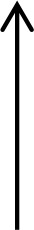	TLR4 inflammatory pathway; Gut barrier; SCFA production; SCFA/GPR41 signaling pathway; Leptin-AMPK/STAT3 signaling pathway	(Xu Y. et al., 2020; Xu et al., 2021)
	*Astragalus* Polysaccharide	Male C57BL/6 J mice	HFD mixture with 4% *Astragalus* polysaccharide for 12 weeks	SCFA-producing bacteria, *Desulfovibrio vulgaris*; *Proteobacteria*, *Epsilonbacteria*	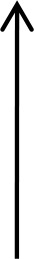	Gut barrier; TLR4/NF-κB signaling pathway; SCFA/GPR41/GPR43 signaling	(Hong et al., 2021; Zhong M. et al., 2022)
	*Lycium barbarum* polysaccharide	Male Sprague–Dawley rats	HFD with *Lycium barbarum* polysaccharide, 50 mg·kg^-1^ ·d^-1^ for 8 weeks; HFD was fed for 18 weeks, and *Lycium barbarum* polysaccharide was administered from the eleventh week	SCFA-producing bacteria *Marvinbryantia*, *Lachnospiraceae NK4A136 group*, *Butyricicoccus* Enterococcaceae	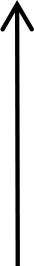 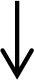	Gut barrier; SCFA production; LPS production; LPS/TLR4/NF-κB signaling pathway; AMPK inflammatory pathway; Lipid metabolism	(Gao et al., 2021; Yang M. et al., 2021; Yang Y. et al., 2021)
	*Platycodon grandiflorum* neutral polysaccharides	Male C57BL/6 mice	Fed a HFD with *Platycodon grandiflorum* neutral polysaccharides, 300 mg/kg/day for 14 weeks	Firmicutes/Bacteroides ratio; *Desulfobacterota*	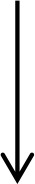	LPS inflammatory pathway; Gut barrier	(Song et al., 2023)
	*Ganoderma lucidum* polysaccharide	Male C57BL/6 J mice	Fed an HFD with *G. lucidum* polysaccharide, 100 or 300 mg/kg/day for 12 weeks	SCFA-producing bacteria *Allobaculum*, *Bifidobacterium Christensenellaceae R-7 group*	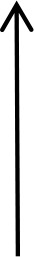	SCFA production; SCFA/GPR43 signaling pathway; LPS production; TLR4/NF-κB signaling pathway; Gut barrier	(Sang et al., 2021)
	Eucommia leaf extract	Male C57BL/6 J mice	HFD with eucommia leaf extract, 320 mg/kg/day for 12 weeks; HFD-(HFD+eucommia leaf extract) FMT after 3 days of antibiotic treatment for 8 weeks	Erysipelotrichaceae; Ruminococcaceae	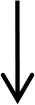 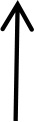	SCFA production; SCFA/GPR41/GPR43 signaling pathway; Gut barrier; Lipid metabolism	(Wang et al., 2023a; Wang et al., 2023b)
	*Penthorum chinense* Pursh. extract	Male C57BL/6J mice	HFD with 2, 4, 8 g/kg/day *Penthorum chinense* Pursh. extract for 8 weeks, HFD was fed for 16 weeks, and *Penthorum chinense Pursh.* extract was administered from the ninth week	*Clostridium IV, Clostridium XIVb, Lactobacillus*	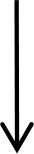	BSH activity; BA and cholesterol metabolism; Gut FXR/FGF15 pathway; Liver FXR	(Li X. et al., 2022)
	ZhiHeShouWu ethanol extract	Male Kunming mice	HFD with ZhiHeShouWu ethanol extract, 0.34, 0,68, or 1.35 g/kg for 5 weeks, HFD was fed for 8 weeks, and ZhiHeShouWu ethanol extract was administered from the fourth week	SCFA-producing bacteria *Phascolarctobacterium*; *Desulfovibrio*	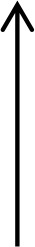 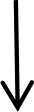	Lipid oxidation; BA metabolism; Gut barrier; Gut FXR/FGFR4 pathway	(Dai et al., 2021)
	*Ganoderma lucidum* ethanol extract	Male Wistar rats	HFD with *G. lucidum* ethanol extract, 150 mg/kg/day for 8 weeks	SCFA-producing bacteria *Alistipes*, Desulfovibrionacea, Peptococcaceae, *Alloprevotella*	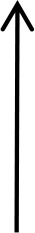	SCFA production; SCFA-GPRs41/43 signaling pathway; Lipid and cholesterol metabolism	(Guo et al., 2018)
	*Phyllanthus emblica* L. aqueous extract	Male C57BL/6J mice	CDAHFD with *Phyllanthus emblica* L. aqueous extract, 0.9, 1.8, or 3.6 g of crude drug/kg for 6 weeks, CDAHFD was fed for 12 weeks, and *Phyllanthus emblica* L. aqueous extract was administered from the seventh week	*Bifidobacterium*, *Alistipes*; Muribaculaceae	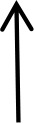 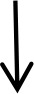	SCFA production; Gut inflammation BA metabolism; Amino acid metabolism	(Luo et al., 2022)
	Ginsenoside extract	Male C57BL/6J mice	HFD with ginsenoside extract, 100 or 200 mg·kg^-1^·d^-1^ for 12 weeks	Muribaculaceae, *Akkermansia*	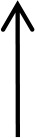	SCFA production; LPS production; Lipid synthesis and metabolism; NF-κB/IκB signaling pathway	(Liang et al., 2021)
	Blueberry extract	Male C57BL/6J mice	Fed with HFD; at the same time, 0.5% (m/v) blueberry extract was added to water for 15 weeks	*Akkermansia*, *Lactobacillus*, *Bifidobacterium*; *Desulfovibrio*	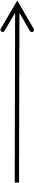 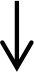	BA metabolism; BSH activity; Gut barrier; FXR/SHP/SREBP-1c signaling pathway;Lipid synthesis;	(Guo et al., 2019)
	Jamun fruit extract	Male C57BL/6J mice	Fed with HFD; at the same time, jamun fruit extract, 100 mg·kg-1·d-1 was orally gavaged for 8 weeks	*Bacteroides*, *Prevotella*, *Alloprevotella*; *Clostridium XlVb*	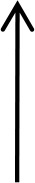 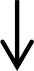	SCFA production; Hepatic lipid synthesis; Insulin resistance	(Xu et al., 2019)
	Camu camu extract	Male C57BL/6J mice	Fed with HFHS; at the same time, 200 mg/kg of resuspended crude extract of camu camu was administered for 8 weeks	*Akkermansia muciniphila*, *Bifidobacterium*, *Barnesiella*; *Lactobacillus*;	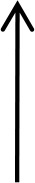 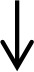	BA metabolism; LPS production; Gut inflammation; Energy consumption; Brown adipose tissue conversion	(Anhê et al., 2018)
	Guava polysaccharides	Male C57BL/6 mice	Fed with HFD; at the same time, guava polysaccharide, 100 mg·kg^-1^·d^-1^ was administered for 11 weeks	*Clostridium XlVa*, *Enterorhabdus*, *Parvibacter* genera; *Mucispirillum*	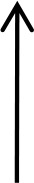 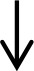	SCFA production; Insulin resistance; Hepatic lipid accumulation; Hepatic inflammation	(Li Y. et al., 2022)
	Noni fruit polysaccharide	Male Sprague-Dawley rats	HFD fed with noni fruit polysaccharide, 100 mg·kg-1 ·d-1 for 5 weeks; HFD was fed for 9 weeks, and noni fruit polysaccharide was administered from the fifth week	*Eubacterium coprostanoligenes*, *Lactobacillus*, *Ruminococcaceae UCG 014*, *Parasutterella*, *Ruminococcus 1*; *Prevotella 9*, *Collinsella, Bacteroides*	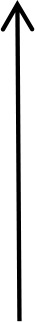 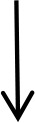	SCFA production; LPS production; Hepatic oxidative stress and inflammation; Colonic epithelium permeability	(Yang X. et al., 2020)
	Polygala japonica Houtt. aqueous extract	Male C57BL/6J mice	Fed with MCD; at the same time, Polygala japonica Houtt. aqueous decoction equivalent to 20 g/kg, 40 g/kg, and 80 g/kg of the crude drug was administered for 7 weeks	*Dubosiella*, *Akkermansia*, *Turicibacter*; *Faecalibaculum*, *Lachnospiraceae NK4A136 group*	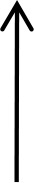 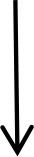	Oxidative stress; Liver inflammation; Tryptophan metabolism; Histidine metabolism	(Liao et al., 2023)
	Quzhou Fructus Aurantii extract	Male C57BL/6J mice	Fed with HFD; at the same time, 300 mg·kg-1·d-1 Quzhou Fructus Aurantii extract was orally gavaged for 12 weeks	*Akkermansia*, *Alistipes*; *Dubosiella*, *Faecalibaculum*, *Lactobacillus*	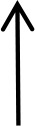 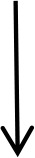	LPS production; TLR4/NF-κB signaling pathway; Gut inflammation; Gut barrier	(Bai et al., 2019)

MAFLD, metabolic-associated fatty liver disease; HFD, high-fat diet; HCD, high-cholesterol diet; MCD, methionine/choline-deficient diet; HFD-DSS, HFD supplemented with 1% dextran sulfate sodium in drinking water; CDAHFD, choline deficient, L-amino acid-defined, high-fat diet with 0.1% methionine; HFHS, high-fat high-sucrose diet; BA, bile acid; SCFA, short-chain fatty acid; LPS, lipopolysaccharide; TMAO, trimethylamine-N-oxide; BSH, bile salt hydrolase; CD36, platelet glycoprotein 4; FXR, farnesoid X receptor; TLR4, Toll-like receptor-4; TGR5, Takeda G-protein-coupled receptor 5; GPR41/GPR43, mammalian G protein-coupled receptors 41/43; NF-κB, nuclear factor kappa-B; TNF-α, tumor necrosis factor alpha; Nrf2, nuclear factor erythroid 2-related factor 2; LXR-α, liver X receptor alpha; SREBP-1c, sterol regulatory element binding protein-1c; AMPK, adenosine 5’-monophosphate (AMP)-activated protein kinase; MyD88, myeloid differentiation primary response protein 88; FGF15, recombinant fibroblast growth factor 15; STAT3, signal transducer and activator of transcription 3; SHP, small heterodimer partner; mTOR, mammalian target of rapamycin; FGFR4, fibroblast growth factor receptor 4.

"↑" indicates an increase in the relative abundance of bacteria; "↓" indicates a decrease in the relative abundance of bacteria.

### 3.1 Plant natural compounds ameliorate MAFLD by regulating the gut microbiota

**Figure 2:**
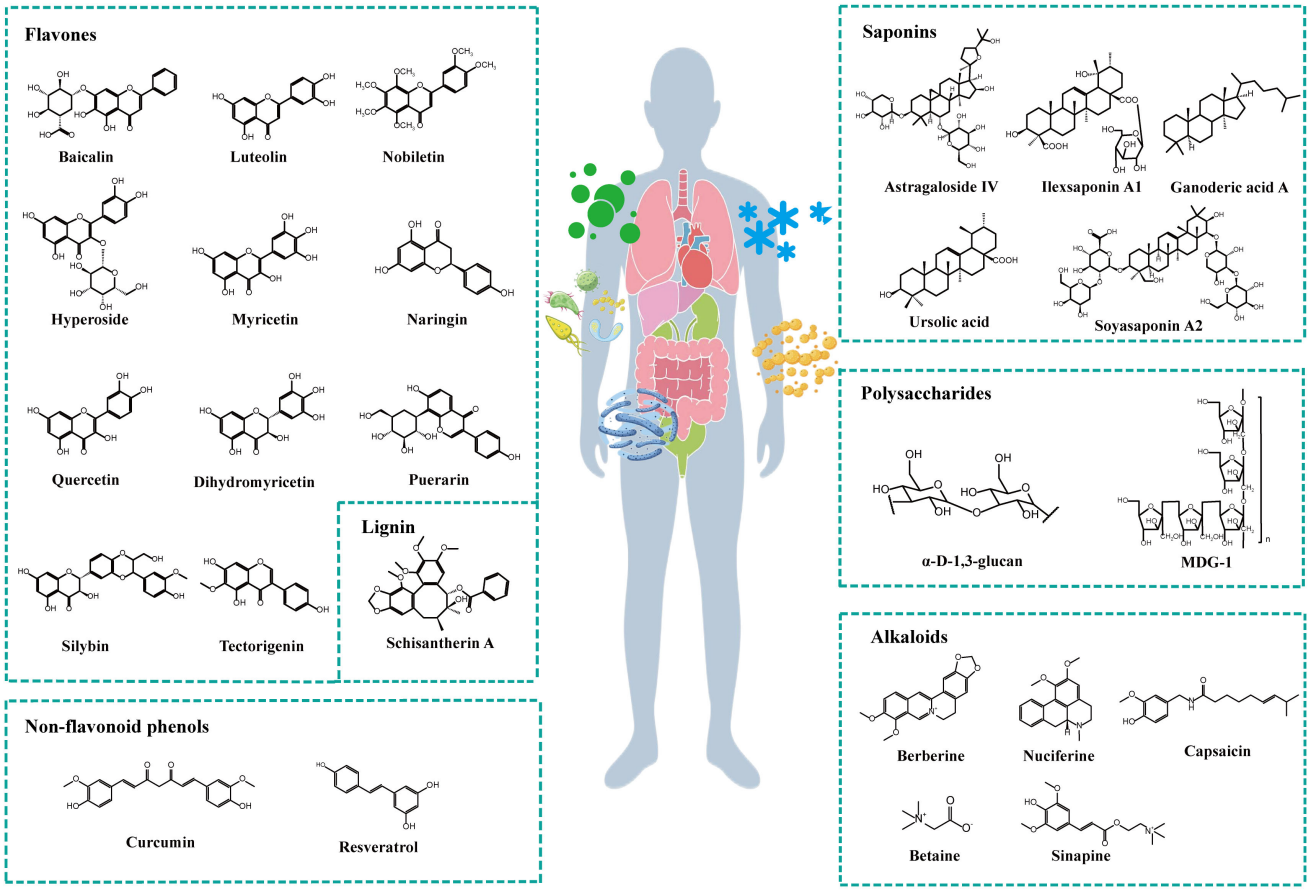
Chemical structures of plant natural compounds.

Plant natural compounds mainly include active ingredients, such as polyphenols, lignans, alkaloids, saponins and polysaccharides. Polyphenols can be divided into flavonoid and nonflavonoid phenolics. Modern pharmacological studies have isolated the numerous active monomeric compounds mentioned above, as shown in **Figure 2**, and have shown that these active monomers can act as therapeutic agents for MAFLD by modulating the gut microbiota and its metabolites.

#### 3.1.1 Polyphenols

##### 3.1.1.1 Flavones

Baicalin is the main active component of the Chinese herb *Scutellaria baicalensis* Georgi. Recent studies have shown that baicalin is a typical multitarget and multipathway drug with hepatoprotective, antitumor, antibacterial, anti-inflammatory, antidepressant and antioxidant effects. Studies suggest that the effect of baicalin in treating MAFLD may be related to the gut microbiota. Baicalin inhibits liver fibrosis and inflammation by increasing the relative abundance of lactobacilli (Liu et al., 2022). Moreover, baicalin increases the abundance of SCFA-producing bacteria, especially butyric acid-producing bacteria, which is beneficial for improving gut homeostasis (Ju et al., 2019). On the other hand, baicalin may reduce hepatic cholestasis and alleviate cholestasis-induced liver fibrosis by increasing the activity of BA-metabolizing enzymes and key receptors such as FXR and TGR5 through targeting of the gut microbiota (Hu et al., 2021).

Luteolin (3’,4’,5,7-tetrahydroxy flavone) is a natural flavonoid that is widely found in vegetables, fruits, and natural herbs and has anti-inflammatory, antioxidant, antitumor and immunomodulatory effects. Recent studies have shown that luteolin intervention markedly regulates the gut microbial composition in MAFLD rats by decreasing the abundances of Desulfovibrionaceae and Coriobacteriaceae to reduce plasma LPS concentrations, inhibiting the TLR4/nuclear factor kappa-B (NF-κB) signaling pathway in the liver, and reducing the secretion of proinflammatory factors (Liu X. et al., 2021). Moreover, luteolin can increase the abundances of *Lactobacillus* and *Bifidobacterium*. Therefore, luteolin may alleviate MAFLD by restoring the microbiota imbalance.

Hyperoside (quercetin-3-O-galactoside) is a flavonol glycoside found in *Crataegus pinnatifida* Bunge., *Artemisia capillaris* Thunb., and *Hyperlcurn perforatum* L. that has anti-inflammatory, hepatoprotective, and antioxidant protective effects. Recent studies have shown that hyperoside can alter the high-fat diet (HFD)-induced gut microbiota composition. Hyperoside modulates the BA composition by inhibiting gut microbes involved in BSH activity, which increases hepatic FXR receptor activation, promotes free fatty acid β-oxidation, and inhibits *de novo* fatty acid synthesis (Wang S. et al., 2021). Therefore, the regulation of BA synthesis and transport by hyperoside may be the mechanism underlying the alleviation of MAFLD.

Myricetin (3,3,4,5,5,7-hexahydroxyflavone) is a flavonoid with antioxidant, anti-inflammatory, antibacterial, anticancer, antidiabetic and hepatoprotective effects (Song et al., 2020). Myricetin supplementation not only increases the abundance of SCFA-producing bacteria to reduce liver lipid synthesis and liver inflammation in HFD-induced MAFLD but also modulates insulin resistance by increasing the fecal butyric acid concentration, which may also benefit the treatment and prevention of MAFLD (Sun et al., 2021).

Quercetin (3,3,4’,5,7-pentahydroxyflavone) is an abundant polyphenolic flavonoid with a variety of bioactivities including antioxidant, anti-inflammatory, antiapoptotic, immunoprotective and anticancer properties. Quercetin can improve gut microbiota imbalance (Wang T. et al., 2023) and modulate gut microbiota metabolites to ameliorate MAFLD. On the one hand, quercetin maintains lipid homeostasis and reduces hepatic steatosis by regulating gut BA metabolism and activating FXR and TGR5 in the liver (Yang et al., 2019; Chen et al., 2021). On the other hand, quercetin can reverse gut microbiota disorders and inhibit the endotoxemia-mediated TLR-4 pathway, which in turn inhibits inflammatory vesicle responses and reticulostriatal pathway activation, leading to blocked lipid metabolism abnormalities (Porras et al., 2016).

Dihydromyricetin, the most abundant flavonoid in *Ampelopsis grossedentata* (Hand.-Mazz.) W.T. Wang (vine tea), has been proven to have anti-inflammatory, antioxidant, antihypertensive, hepatoprotective, lipid-modulating and antitumor effects. Studies have indicated that dihydromyricetin can prevent and ameliorate MAFLD by regulating the gut microbiota and its metabolites. Dihydromyricetin not only affects BSH activity by reducing the abundance of *Lactobacillus* but also enhances BA binding and BA transport in the liver and inhibits FXR-related signaling pathway-mediated BA reabsorption in the ileum. In addition, dihydromyricetin can improve gut mucosal barrier function; increase the expression of gut tight junction proteins such as zonula occludens protein 1 (ZO-1), Occludin, and Claudin1; improve gut microvillus structure; and reduce peripheral serum LPS levels (Tong, 2018).

Silybin is isolated from the seeds of *Silybum marianum* (L.) Gaertn. (milk thistle) and is widely used as a hepatoprotective agent (Federico et al., 2017). Studies suggest that the effect of silybin in treating liver diseases may be achieved by modulating the gut microbiota and its metabolites (Li X. et al., 2020). Mechanistic studies revealed that silybin supplementation increased the relative abundances of the SCFA-producing *Blautia*, *Bacteroides* and *Akkermansia*, thereby increasing the gut levels of acetate, propionate and butyrate while suppressing the levels of formate, ultimately exhibiting protective effects against MAFLD and hepatitis. In addition, silybin improved gut mucosal barrier function by increasing ZO-1 and Occludin expression. However, silybin also inhibits some known beneficial bacteria, such as *Alloprevotella* and *Lactobacillus*; therefore, the relationship between silymarin-induced alterations in the gut microbiota and the alleviation of MAFLD needs to be investigated in greater depth.

Tectorigenin, a methoxy isoflavone with three hydroxyl groups, can be isolated from many medicinal plants, such as *Iris unguicularis*, *Belamcanda chinensis* (L.) DC., and *Pueraria lobata* (Willd.) Ohwi. Modern medical studies have shown that tectorigenin has anti-inflammatory, antioxidative, and antidiabetic effects on oxidative stress injury. Duan et al. (Duan et al., 2022) reported that tectorigenin could restore gut barrier function by promoting the growth of *Akkermansia* and inhibiting *Turicibacter*, *Dubosiella* and *Faecalibaculum*, which in turn reduced LPS levels and inhibited the LPS/TLR-4/NF-κB/tumor necrosis factor alpha (TNF-α) pathway to alleviate liver inflammation. Furthermore, tectorigenin can also activate the expression of hepatic and gut FXR, which is involved in the reduction of serum TBA levels and the excretion of fecal BAs, to reduce lipid accumulation and bacterial translocation. Therefore, tectorigenin may potentially mediate the gut-liver axis to attenuate MAFLD.

Puerarin (7,4-dihydroxy-isoralone-8-glucoside) is an active isoflavone glycoside extracted from the dry root of *P. lobata* that has vasodilatory, cardioprotective, antioxidant, anti-inflammatory, and anti-insulin resistance effects. It was found that puerarin treatment could inhibit the activity of the LPS-producing *Helicobacter* species, promote the activity of the butyrate-producing *Roseburia* species, and increase the abundance of *Akkermansia muciniphila* (Gong et al., 2021) to increase gut expression levels of Mucin 2 and Reg3g and protect gut barrier function by increasing ZO-1 and Occludin expression *in vivo* and *in vitro* (Wang L. et al., 2019). Thus, the anti-MAFLD activity of puerarin may be closely associated with the regulation of the gut microbiota.

Nobiletin is a polymethoxyflavonoid that is the main component of Citri Reticulatae Pericarpium and has been shown to have anti-obesity (Lee et al., 2012), antihyperglycemic (Lee et al., 2010), antihypercholesterolemic and anti-MAFLD activities (Kim et al., 2021). Recently, Li et al. (Li et al., 2023) reported that nobiletin treatment not only ameliorated high-fat high-sucrose feed-induced lipid accumulation but also reversed the dysbiosis of the gut microbiota in mice with MAFLD and increased the relative abundance of *Allobaculum stercoricanis* and *Lactobacillus casei*. Moreover, untargeted metabolomics revealed that nobiletin modulated the metabolism of the long-chain fatty acid myristoleic acid and experimentally demonstrated the protective effects of fecal transplantation as well as the administration of bacteria or metabolites to treat MAFLD. These results suggest that nobiletin may be a potential treatment for MAFLD, but further studies are needed.

Naringin, a naturally occurring flavonoid found predominantly in the rinds of grapefruit and citrus fruits, has been reported to have antihyperglycemic (Ahmed et al., 2017) and antihyperlipidemic properties (Yu X. et al., 2022). Mu et al. (Mu et al., 2020) reported that naringin treatment increased the relative abundance of *Allobaculum*, *Alloprevotella*, *Butyricicoccus* and *Parasutterella* and downregulated the expression of the sterol regulatory element binding protein 1, fatty acid synthase, acetyl-CoA carboxylase 1, and stearoyl-CoA desaturase 1 proteins, effectively attenuating hepatic *de novo* fatty acid synthesis. On the other hand, naringin treatment reduced the relative abundance of the deleterious *Campylobacte*r species and the proinflammatory *Faecalibaculum* and *Fusobacterium* species, which may be associated with the reduction in LPS levels in the serum and hepatic inflammatory cells of mice with MAFLD. Thus, naringin may attenuate NAFLD by preventing gut ecological dysregulation, but experimental evidence confirming that naringin attenuates MAFLD by directly modulating the gut microbiota is lacking.

##### 3.1.1.2 Nonflavonoid phenols

Curcumin is a natural polyphenol compound isolated from *Curcuma longa* L. that has been proven to have antiobesity, anticancer, antioxidant, hepatoprotective and other biological activities. Studies suggest that curcumin may ameliorate MAFLD by regulating the gut microbiota through the enrichment of the SCFA-producing bacteria *Bacteroides*, *Parabacteroides*, *Alistipes* and *Alloprevotella* and a reduction in the abundance of the endotoxin-producing *Desulfovibrio* (Li S. et al., 2021). In addition, curcumin can inhibit hepatic lipogenesis and promote BA metabolism by regulating the nuclear factor erythroid 2-related factor 2 (Nrf2)/FXR/liver X receptor alpha (LXR-α) pathway (Yan et al., 2018). Furthermore, the results of a comparative study of curcumin and metformin showed that they had similar effects in reducing hepatic steatosis, improving gut barrier integrity and regulating the gut microbiota in rats with HFD-induced obesity and that curcumin may prove to be a novel adjuvant therapy for MAFLD (Li R. et al., 2020).

Resveratrol is a natural polyphenolic compound that is mainly isolated from *Vitis vinifera* L. (grape) and has anticancer, anti-inflammatory, antioxidant, and antiobesity effects. Resveratrol can attenuate HFD-induced steatosis through modulation of the gut microbiota composition (Wang et al., 2020a; Wang P. et al., 2019; Wang et al., 2020b; Yin et al., 2020). Resveratrol not only reduced the relative abundances of the harmful bacteria *Desulfovibrio*, *Lachnospiraceae NK4A316 group* and *Alistipes* but also increased the relative abundances of the SCFA-producing bacteria *Allobaculum* and *Blautia* (Wang et al., 2020b) and the gut metabolites 4-hydroxyphenylacetic acid and 3-hydroxyphenylpropionic acid, contributing to the improvement in lipid metabolism (Wang et al., 2020a). Furthermore, transplantation of the resveratrol-induced microbiota into HFD-fed mice also showed therapeutic effects (Wang et al., 2020a; Wang P. et al., 2019; Wang et al., 2020b; Yin et al., 2020). These results demonstrated that resveratrol has the potential to regulate the gut-liver axis to ameliorate MAFLD.

#### 3.1.2 Lignin

Schisantherin A is an active substance isolated from the fruit of *Schisandra chinensis* (Turcz.) Baill., a perennial deciduous woody liana with antiparkinsonian, anti-inflammatory, hepatoprotective, ischemia–reperfusion injury prevention and osteoclast formation inhibition effects (Xiao et al., 2022). The hepatoprotective effects of schisantherin A may be achieved by improving gut inflammation and modulating the gut microbiota (Yu S. et al., 2022). On the one hand, schisantherin A treatment alleviated the HFD-induced imbalance in the gut microbiota by reducing the abundance of Firmicutes and increasing the abundance of Bacteroidetes. Furthermore, antibiotic treatment demonstrated the role of the gut microbiome in the schisantherin A-mediated improvement in liver inflammation. On the other hand, schisantherin A treatment reduced gut LPS production and release in HFD-fed mice and inhibited the LPS-TLR4 signaling pathway to ameliorate gut permeability impairment and inhibit the progression of MAFLD to NASH.

#### 3.1.3 Alkaloids

Berberine, also known as safranin, is isolated from the root of *Coptis chinensis* Franch. and is traditionally used to treat diarrhea. Modern pharmacological studies have reported that berberine is beneficial for treating metabolic disorders, such as type 2 diabetes, dyslipidemia, and MAFLD/NASH. However, because the bioavailability of berberine is low, the gut microbiome and microbe-derived metabolites are thought to be key factors involved in the mechanisms of action of berberine (Cheng et al., 2021). Berberine may exert its lipid-lowering effects mainly through the regulation of BA turnover and thus through the gut FXR signaling pathway (Sun et al., 2016; Shu et al., 2021). Sun et al. reported that berberine increased taurine-coupled BA levels by reducing the relative abundance of BSH-producing bacteria, which activated the gut FXR pathway (Sun et al., 2016). Shu et al. reported that berberine alleviated NASH by modulating the gut microbiota and BA metabolism and upregulating gut FXR and recombinant fibroblast growth factor 15 (FGF15) expression and FGF15 secretion to further inhibit adipogenesis and NF-κB activation in the liver (Shu et al., 2021).

Nuciferine, the major functional aporphine alkaloid from the dried leaves of *Nelumbo nucifera* Gaertn., has been shown to be useful for reducing body weight, lowering serum and liver lipids, and alleviating hepatic steatosis and liver damage. Nuciferine supplementation not only reduced the abundances of BSH-producing and 7α-dehydroxylated bacteria, leading to the accumulation of coupled BAs as FXR antagonists to inhibit gut FXR signaling but also regulated the BA cycle *in vivo* by modulating the levels of the rate-limiting enzymes CYP7A1 and cytochrome P450 27A1 (CYP27A1) and the BA transporters bile salt export pump (BSEP) and Na^+^-taurocholate cotransporting polypeptide (NTCP) in the liver (Sun et al., 2022). In addition, nuciferine elevated the relative abundances of mucus-associated microbes (*Akkermansia muciniphila* and Ruminococcaceae) and SCFA-producing bacteria to improve gut barrier integrity and reduce liver inflammation by decreasing the abundance of LPS-producing microbes (Desulfovibrionaceae), reducing LPS production, and inhibiting the TLR4/myeloid differentiation primary response protein 88/NF-κB signaling pathway (Fan et al., 2022; Wang Y. et al., 2020).

Capsaicin (trans-8-methyl-N-vanillyl-6-nonenamide) is one of the active ingredients of *Capsicum annuum* L. (pepper) and has anti-statogenic, antioxidant, anti-inflammatory and antifibrotic effects. Studies have indicated that capsaicin treatment increases the relative abundances of *Bacteroidales S24-7*, *Akkermansia* and *Allobaculum*, leading to the accumulation of SCFAs, which in turn enhances lipid accumulation and decreases TG and TC levels. Furthermore, capsaicin decreased BSH activity by inhibiting *Lactobacillus*, which increased the levels of conjugated BAs, especially tauro-β-muricholic acid (T-β-MCA), which in turn inhibited the enterohepatic FXR-FGF15 axis to regulate the composition of the BA pool (Gong et al., 2022). These results suggested that capsaicin can mitigate MAFLD by regulating the gut microbiota and the composition of BAs and SCFAs.

Betaine is a quaternary ammonium-type water-soluble alkaloid with antioxidant and anti-inflammatory effects. Betaine is an amino acid (trimethylglycine) that is a necessary intermediate in the catabolism of choline. Betaine supplementation can increase the abundances of *Akkermansia muciniphila*, *Lactobacillus* and *Bifidobacterium* and promote the production of SCFAs (Du et al., 2021). In mice lacking a gut microbiota, the ability of betaine to prevent HFD-induced obesity and metabolic syndrome was significantly reduced, which suggested that betaine can alleviate MAFLD by regulating the gut microbiota. In addition, Wang et al. (Wang F. et al., 2019) reported that betaine can reduce liver lipid accumulation by improving gut BA and TMA-related oxidative metabolism in blunt mouth bream.

Sinapine accounts for 70-80% of rapeseed polyphenols and may have the potential to ameliorate MAFLD. It was shown that sinapine intervention could regulate the HFD-induced gut microbiota, increase the relative abundance of the probiotic bacteria Lactobacillaceae, and inhibit endotoxin production by reducing the relative abundance of *Desulfovibrio*. Moreover, supplementation with sinapine could increase the abundance of the SCFA-producing bacteria Akkermansiaceae and *Blautia*, which increase SCFA levels and upregulate the GPR43 receptor to inhibit gut inflammatory factor expression (Li Y. et al., 2019). Therefore, sinapine has a therapeutic effect on MAFLD by regulating the gut microbiota.

#### 3.1.4 Saponins

Astragaloside IV is one of the major saponin compounds extracted from the roots of *Astragalus membranaceus* (Fisch.) Bge. and has anti-inflammatory, anti-liver fibrosis, antioxidative stress, anti-asthma, antidiabetic, immunomodulatory and cardioprotective effects (Li et al., 2016). Astragaloside IV inhibits gut FXR expression by reducing the abundance of BSH-expressing bacteria and decreasing BSH activity, thus increasing the level of T-β-MCA, which is often accompanied by a decrease in FGF15 and subsequent activation of hepatic FXR, leading to the inhibition of hepatic steatosis (Zhai et al., 2022). Moreover, fecal transplantation experiments further demonstrated that the action of astragaloside IV is dependent on the gut microbiota. Therefore, alterations in the gut microbiota and BAs may be involved in the mechanism of action of astragaloside IV for treating MAFLD.

Ilexsaponin A_1_ is one of the most abundant triterpenoid saponins and has antithrombotic and anticoagulant properties; it was isolated from *Ilex chinensis* Sims, a small evergreen tree with red fruit. Previous studies have shown that the combination of ilexhainanoside D and ilexsaponin A_1_ modulates the gut microbiota, restores gut barrier function, and ameliorates gut inflammation (Zhao et al., 2019). A separate study of ilexsaponin A_1_ revealed that ilexsaponin A_1_ may exert cholesterol-lowering and MAFLD-inhibiting effects by altering the gut microbiota and BA metabolism (Zhao et al., 2021). Ilexsaponin A_1_ intervention enhanced BSH activity by increasing the relative abundance of BSH-producing bacteria, which increased BA uncoupling and excretion in the ileum. Moreover, ilexsaponin A_1_ intervention also increased FXR and BSEP expression and decreased NTCP expression, which increased hepatic BA efflux and reduced BA uptake. In addition, intake of ilexsaponin A_1_ also decreased the serum LPS content and the expression of inflammatory cytokines.

Ganoderic acid A is one of the most abundant triterpenes isolated from the red fungus *Ganoderma lucidum* (Leyss. ex Fr.) Karst. and has antinociceptive, antioxidative and anticancer pharmacological effects. Ganoderic acid A intervention increased the relative abundances of *Eisenbergiella tayi*, *Alistipes senegalensis*, *Oscillibacter valericigenes*, *Bacteroides acidifaciens*, *Mucispirillum schaedleri* and *Bacteroides eggerthii* but significantly reduced the relative abundances of *Parabacteroides goldesteinii*, *Anaerotruncus colihominis*, *Barnesiella intestinihominis*, and *Lactobacillus murinus*, which increased the production of SCFAs in the gut. In addition, ganoderic acid A treatment can interfere with the regulation of BAs. Ganoderic acid A not only upregulated the expression of liver genes (FXR, CYP7A1 and NTCP) involved in BA homeostasis and reduced liver BA levels but also promoted the excretion of BAs through the feces (Guo et al., 2020). Therefore, the ability of ganoderic acid A to improve MAFLD may be mediated by regulation of the gut microbiota and metabolites.

Ursolic acid is a natural pentacyclic triterpene carboxylic acid that occurs naturally in various fruits and vegetables, such as apples, blueberries and cranberries, and has antioxidant, anti-inflammatory, anticancer and hepatoprotective effects. Recent studies have indicated that the addition of ursolic acid is effective at promoting the growth of *Bifidobacterium*, which is recognized as a genus of SCFA-producing bacteria. *Bifidobacterium* species, as recognized probiotics, have been shown to be effective at limiting the production of the endotoxins LPSs. In addition, ursolic acid can promote the production of SCFAs, including acetic acid, propionic acid and butyric acid, in feces (Hao et al., 2020). Therefore, ursolic acid holds promise as a potential therapeutic agent for ameliorating MAFLD, but further studies are needed.

Soyasaponins are phytochemicals found in *Glycine max* (L.) Merr. (soya bean) and that have antioxidant, anti-inflammatory, hypoglycemic, cholesterol-lowering, and hepatoprotective effects. Soyasaponin A_2_ is a monomer of soyasaponins, and Xiong et al. (Xiong et al., 2021) reported that the hepatoprotective effect of soyasaponin A_2_ against NASH may be achieved by modulating the gut microbiota metabolite BAs. On the one hand, soyasaponins A_2_ may alleviate methionine–choline-deficient (MCD) diet-induced steatohepatitis by reducing the abundance of Erysipelotrichaceae and *Faecalibaculum* species. On the other hand, it may not only effectively block gut-liver circulation and the reabsorption of BAs in the terminal ileum by forming mixed micelles with BAs but also directly bind to BAs and promote their excretion via feces.

#### 3.1.5 Polysaccharides

*Radix Puerariae thomsonii*, the root of the botanical family *Fabaceae* species *Pueraria montana* var. *thomsonii* (Benth.) MR Almeida, can be used as food or medicine. Recently, an α-D-1,3-glucan isolated and purified from *Radix Puerariae thomsonii* was shown to not only reduce HFD-induced liver injury, inflammation, glucose metabolism, and steatosis but also regulate the gut microbiota and its metabolites (Li Q. et al., 2022). α-D-1,3-glucan treatment not only increased the relative abundance of *Lactobacillus*, *Phocea*, *Ruthenibacterium*, *Flavonifractor*, *Oscillabacter*, *Flavinibacter*, and *Butyricoccus* to increase butyric acid, propionic acid and acetate levels but also promoted the integrity of the gut barrier by increasing the expression of tight junction proteins (ZO-1, Occludin, and Claudin-4) and mucin (Mucin 2). In addition, α-D-1,3-glucan administration significantly reduced the serum levels of the proinflammatory factor TNF-α and LPSs and inhibited HFD-induced inflammation. In conclusion, α-D-1,3-glucan is expected to be a potential drug for alleviating MAFLD.

MDG-1, a β-D-fructose polysaccharide isolated and purified from the root of *Ophiopogon japonicus* (L.f.) Ker-GawL., prevents HFD-induced obesity and hyperlipidemia in mice. MDG-1 modulates the structure of the gut microbiota by promoting the growth of beneficial SCFA-producing bacteria and reducing the abundance of endotoxin-producing pathogens to inhibit the inflammatory response and liver lipid metabolism (Wang X. et al., 2019). In addition, MDG-1 intervention markedly promoted the growth of *Akkermansia muciniphila*, the abundance of which was inversely proportional to MAFLD (Zhang L. et al., 2021). Therefore, MDG-1 may be a potential agent for preventing and treating MAFLD by targeting the gut microbiota.

### 3.2 Herb extracts ameliorate MAFLD by regulating the gut microbiota

**Figure 3:**
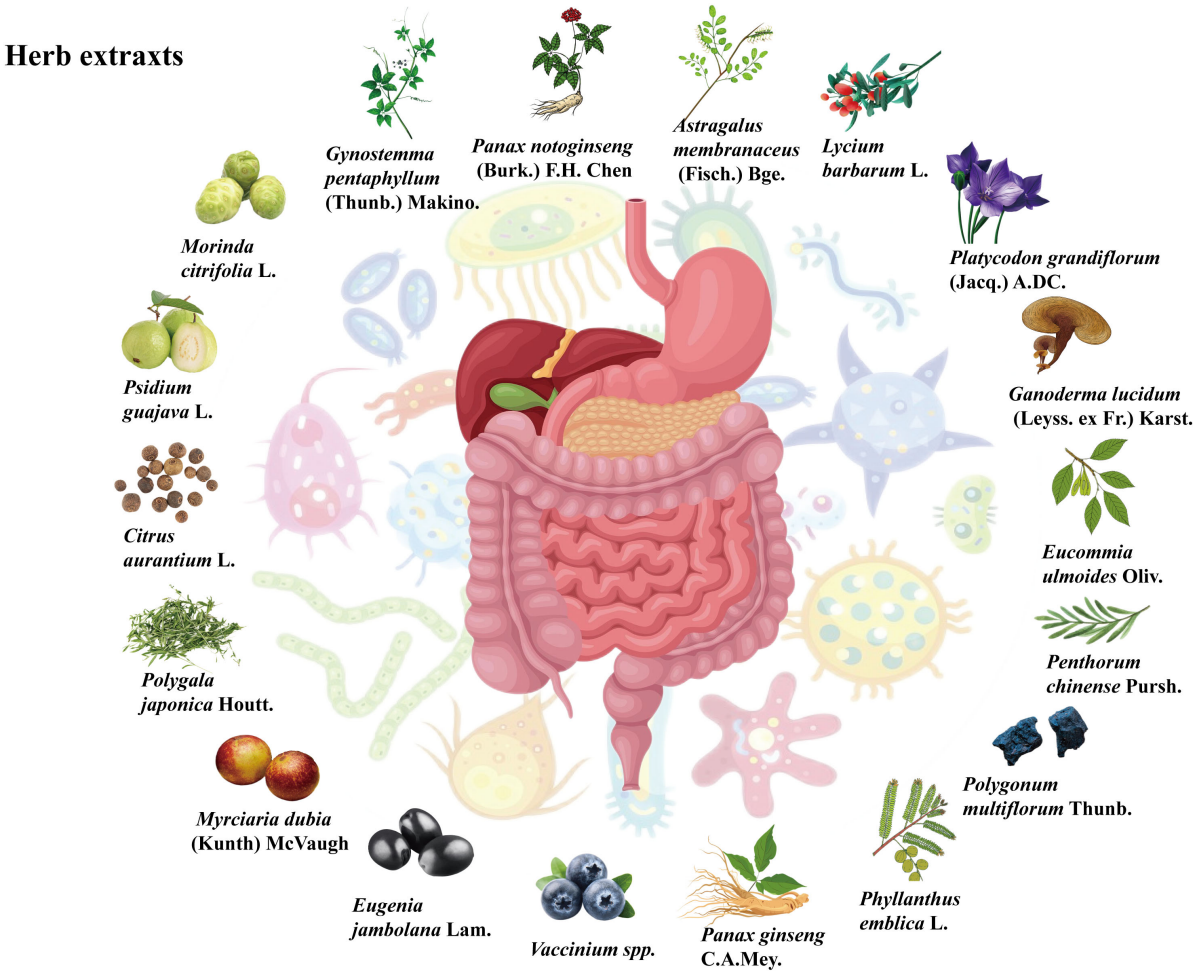
Sources of the herb extracts.

Plant natural products also include extracts with diverse functions, including total polyphenols, total saponins, and total polysaccharides. Plant natural products may act through a single compound in the total extract, but this phenomenon has not been studied at the monomer level because of certain limitations, such as those of isolation techniques. On the other hand, the complex compounds of the extracts may interact with each other and work together to exert therapeutic effects. Studies have shown that some herbal extracts can alter the gut microbiota and its metabolites to exert beneficial effects against MAFLD, as shown in **Figure 3**.

Gypenosides are the main components of *Gynostemma pentaphyllum* (Thunb.) Makino, a herb known as “southern ginseng”. These compounds are widely used as hepatoprotective agents in Asia, and their mechanism of action may be related to improvement of the gut microbiota. Gypenosides not only promoted the growth of SCFA-producing bacteria (*Akkermansia*, *Bacteroides* and *Parabacteroides*) and increased the content of SCFAs (acetate, propionate and butyrate) but also decreased the relative abundances of harmful bacteria (*Desulfovibrio*, *Escherichia-Shigella* and *Helicobacter*) to inhibit LPS production, thereby suppressing inflammation (Zhong F-W. et al., 2022). In addition, gypenosides also inhibited hepatic lipid synthesis by modulating the gut BA composition and increasing hepatic FXR and SHP levels (Li H. et al., 2020).

*Panax notoginseng* saponins are the main bioactive components of *Panax notoginseng* (Burk.) F.H. Chen, also known as “the king of ginseng”, and have been found to reduce serum lipid and liver lipid accumulation. *Panax notoginseng* saponins promoted the growth of *Akkermansia muciniphila* and *Parabacteroides distasonis*, which activated the leptin-AMPK/signal transducer and activator of transcription 3 signaling pathway to promote energy expenditure and reduce lipid accumulation (Xu Y. et al., 2020). In addition, *Panax notoginseng* saponin supplementation can increase the levels of long-chain fatty acids (oleic acid and 2-palmitoyl glycerol) and SCFAs (acetic acid, butyric acid, propionic acid, etc.) in feces, and then large amounts of SCFAs enter the liver to activate GPR41, further ameliorating steatosis and inflammation (Xu et al., 2021). Therefore, *Panax notoginseng* saponins may alleviate MAFLD by regulating the structure and function of the gut microbiota.

*Astragalus* polysaccharide is an active ingredient isolated from *A. membranaceus* that has antioxidant, anti-inflammatory, hypoglycemic, antiviral, hypolipidemic and immunomodulatory effects. Mechanistic studies suggest that *Astragalus* polysaccharide may ameliorate MAFLD by altering the gut microbiota composition. Hong et al. (Hong et al., 2021) reported that *Astragalus* polysaccharide can specifically enrich SCFA-producing bacteria, such as *Desulfovibrio vulgaris*, which can effectively produce acetic acid and inhibit the expression of liver fatty acid synthase and platelet glycoprotein 4 protein to exert anti-MAFLD effects. Zhong et al. (Zhong M. et al., 2022) reported that Mongolian *Astragalus* polysaccharide intervention could decrease the ratio of Firmicutes to Bacteroides and increase the abundances of *Proteobacteria* and *Epsilonproteobacteria*. In addition, Mongolian *Astragalus* polysaccharide supplementation may alleviate liver inflammation and lipid accumulation in MAFLD treatment through the gut microbiota by modulating TLR4-mediated inflammatory pathways and the SCFA-GPR signaling pathway.

*Lycium barbarum* polysaccharide is the main bioactive component of *Lycium barbarum* L. (goji berry) and has many pharmacological properties, such as serum glucose-lowering, serum lipid-lowering, anti-inflammatory, antioxidant and antitumor effects. Numerous studies have demonstrated that *Lycium barbarum* polysaccharide can alleviate MAFLD by altering the gut microbiota composition and SCFA metabolism (Gao et al., 2021; Yang M. et al., 2021; Yang Y. et al., 2021). *Lycium barbarum* polysaccharide intervention increased the relative abundances of the SCFA-producing bacteria *Marvinbryantia*, *Lachnospiraceae NK4A136 group* and *Butyricicoccus*, which increased the content of fecal SCFAs (Yang M. et al., 2021; Yang Y. et al., 2021). Moreover, it also inhibited the LPS/TLR4/NF-κB signaling pathway by inhibiting the increase in the harmful bacteria Enterococcaceae and their metabolite LPSs (Gao et al., 2021), thereby alleviating MAFLD. Therefore, *Lycium barbarum* polysaccharide is expected to be a promising treatment for MAFLD.

*Platycodon grandiflorum* neutral polysaccharides are active components isolated and purified from *Platycodon grandiflorum* (Jacq.) A.DC. (balloon flower) that have antioxidative stress and immunomodulatory activities. Song et al. (Song et al., 2023) studied mice with HFD-induced obesity and reported that *Platycodon grandiflorum* neutral polysaccharide treatment could reduce the Firmicutes/Bacteroides ratio and inhibit the release of LPSs into the gut by reducing the abundance of *Desulfobacterota*, preventing an inflammatory response and disrupting gut energy metabolism. In addition, *Platycodon grandiflorum* neutral polysaccharides can increase gut tight junction protein expression and ameliorate gut leakage. Although the lipid-lowering effect of *Platycodon grandiflorum* neutral polysaccharides has been confirmed, whether they can prevent MAFLD through the gut microbiota requires fecal microbiota transplantation (FMT) experiments.

*G. lucidum* polysaccharides are the main active components isolated from *G. lucidum* and have immunomodulatory, antioxidant, anti-inflammatory, antitumor, antiobesity and antidiabetic effects. Several studies have reported that *G. lucidum* polysaccharides can prevent obesity and MAFLD by regulating the gut microbiota (Sang et al., 2021). *G. lucidum* polysaccharides increased the relative abundances of SCFA-producing bacteria and potential probiotics, such as *Allobaculum*, *Bifidobacterium* and *Christensenellaceae R-7 group*, which activated the GPR43 receptor in adipose tissue to regulate metabolism and inhibit obesity. In addition, *G. lucidum* polysaccharides inhibited the HFD-induced inflammatory response by reducing the serum LPS concentration and inhibiting the TLR4/NF-κB signaling pathway. Therefore, *G. lucidum* polysaccharide may be a potential agent for targeting the gut microbiota to prevent obesity, hyperlipidemia and MAFLD.

Eucommia, isolated from the dry roots of *Eucommia ulmoides* Oliv., is a Chinese medicine and food homolog that has antiosteoporotic, anti-inflammatory, hypoglycemic and hypolipidemic effects. Recent studies have indicated that Eucommia leaves have similar active ingredients and antihyperlipidemic effects as Eucommia bark and can modulate the gut microbiota to alleviate lipid metabolism disorders (Wang et al., 2023a; Wang et al., 2023b). Eucommia leaf extract supplementation increased the relative abundance of Ruminococcaceae to promote the production of butyric acid, which upregulated the expression of GPR43 to reduce the area of adipocytes and lipid accumulation. Furthermore, it reduced the relative abundance of the harmful Erysipelotrichaceae species. Therefore, Eucommia leaf extract has the potential to alleviate MAFLD by targeting the gut microbiota.

*Penthorum chinense* Pursh. has been widely used as a traditional Chinese functional food to prevent and treat liver diseases. Numerous studies have demonstrated the therapeutic effects of *P. chinense* extract on MAFLD through alterations in the gut microbiota and its metabolite BAs (Li X. et al., 2022). *P. chinense* extract reduced the relative abundances of *Clostridium IV*, *Clostridium XIVb*, and *Lactobacillus* to inhibit BSH activity, inhibited BA uncoupling and dehydroxylation, and increased taurochenodeoxycholic acid (TCDCA) and tauroursodeoxycholic acid (TUDCA) levels. TCDCA and TUDCA, which are naturally occurring FXR antagonists, can lower cholesterol levels by inhibiting gut FXR activity, downregulating FGF15 expression, activating hepatic BA synthase activity, and promoting cholesterol conversion. In addition, *P. chinense* extract increased chenodeoxycholic acid production, which activated hepatic FXR expression and increased hepatic FXR-induced BA excretion levels, thus further promoting the conversion of cholesterol to BAs.

ZhiHeShouWu is obtained by processing *Polygonum multiflorum* Thunb. in black bean juice and is often used clinically to regulate lipid metabolism. Studies have shown that supplementation with ZhiHeShouWu ethanol extract could increase the relative abundances of the SCFA-producing *Phascolarctobacterium* species and reduce the relative abundance of *Desulfovibrio* (Dai et al., 2021). In addition, the ZhiHeShouWu ethanol extract affected BA metabolism by remodeling the gut microbiota, thus inhibiting the expression of gut FXR genes, accelerating cholesterol metabolism *in vivo*, and maintaining cholesterol homeostasis. Therefore, ZhiHeShouWu ethanol extract may improve MAFLD by regulating the gut microbiota composition and maintaining gut barrier function.

*G. lucidum*, used in TCM for thousands of years, has a variety of activities, such as hepatoprotective, antitumor and antiaging activities. As described above for *G. lucidum*, its hepatoprotective effect is associated with the regulation of the gut microbiota. The *G. lucidum* ethanol extract was also able to improve HFD-induced gut microbiota disorders due to its abundance of triterpenoids (Guo et al., 2018). The *G. lucidum* ethanol extract significantly elevated the relative abundances of *Alistipes*, Desulfovibrionaceae, Peptococcaceae, and *Alloprevotella* to increase gut propionic acid and butyric acid levels, which have been associated with positive effects on gut health, such as anti-inflammatory effects and improvement in glucose homeostasis and other metabolic symptoms. Therefore, the mechanism of action of the *G. lucidum* ethanol extract in improving MAFLD may involve regulation of the gut microbiota and SCFAs.

*Phyllanthus emblica* L. is an edible medicinal fruit used to treat hepatobiliary disorders, viral hepatitis, alcoholic hepatitis, MAFLD, and hepatocellular carcinoma. It has been shown that *P. emblica* aqueous extract can improve MAFLD by remodeling the gut microbiota (Luo et al., 2022). On the one hand, *P. emblica* aqueous extract elevated the relative abundances of *Bifidobacterium* and *Alistipes* and reduced the relative abundance of Muribaculaceae. Remodeling of the gut microbiota helped to increase SCFA levels, suppress gut inflammation and maintain gut homeostasis. On the other hand, *P. emblica* aqueous extracts affected choline-deficient, L-amino acid-defined, high-fat diet-induced metabolic disorders by modulating primary BA biosynthesis and taurine and hypotaurine metabolism.

Ginsenosides are the main bioactive components isolated from *Panax ginseng* C.A.Mey. (ginseng) and have many biological effects, such as antiobesity, antihyperglycemia and anti-MAFLD effects. Studies have shown that various isoforms of ginsenosides, such as Rg1 (Hou et al., 2020), Rg2, Rh1 (Wang F. et al., 2021), CK (Zhang J. et al., 2021), Rb2 (Huang et al., 2017), Rc (Yang Z. et al., 2020), and Rf (Chen et al., 2022), have the potential to alleviate MAFLD. Liang et al. (Liang et al., 2021) reported that ginsenoside extract treatment could modulate HFD-induced gut microbiota imbalance and attenuate ecological dysbiosis-mediated gut leakage and metabolic endotoxemia, which may be a potential mechanism of action for improving MAFLD. Ginsenoside extract supplementation elevated the relative abundances of Muribaculaceae and *Akkermansia*, which promoted the production of SCFAs in the gut, thereby providing energy to colonic epithelial cells and improving lipid accumulation and liver inflammation. In addition, ginsenoside extract treatment also reduced the serum LPS concentration and attenuated metabolic endotoxemia in HFD-fed mice.

Blueberries (*Vaccinium* spp.) are rich in polyphenols and are known for their antioxidant and cardiovascular protective properties (Emily et al., 2023). Studies have shown that blueberry extract may ameliorate HFD-induced obesity by modulating the composition of the gut microbiota (Guo et al., 2019). On the one hand, blueberry extract significantly increased the relative abundance of *Akkermansia*, *Lactobacillus*, and *Bifidobacterium*, which increased BSH activity, resulting in lower plasma and liver TG concentrations in mice. In addition, blueberry extract administration significantly reduced plasma taurine-coupled BAs, including tauro-α-muricholic acid (T-α-MCA) and T-β-MCA levels, to activate FXR in the liver, which enhanced SHP expression and further inhibited the activity of SREBP-1c and its downstream genes associated with lipid synthesis. On the other hand, blueberry extract reduced the abundance of *Desulfovibrio* and increased colonic Mucin 2 and ZO-1 levels, thereby attenuating gut permeability. In conclusion, blueberry extract may ameliorate MAFLD by modulating the gut microbiota and bile acid pool.

*Eugenia jambolana* Lam., commonly known as jamun, is an edible berry that has been shown to have antioxidant, anti-inflammatory and hepatoprotective activities (Chhikara et al., 2018). Xu et al. (Xu et al., 2019) found that jamun fruit extract not only ameliorated HFD diet-induced obesity, hepatic steatosis and insulin resistance but also regulated the gut microbiota and SCFA metabolism. Jamun fruit extract supplementation increased the relative abundance of *Bacteroides*, *Prevotella* and *Alloprevotella*, not only promoting the content of total SCFAs and acetic, propionic and butyric acids but also mitigating the imbalance in the major components of SCFAs, especially the propionic/n-butyric acid ratio. In addition, jamun fruit extract also reduced the level of *Clostridium XlVb*, which has been reported to be associated with cognitive impairment in patients with Parkinson’s disease (Qian et al., 2018). In conclusion, jamun fruit extract may ameliorate MAFLD by modulating the gut microbiota and SCFA production.

*Myrciaria dubia* (Kunth) McVaugh, commonly known as camu camu, is an Amazonian fruit rich in vitamin C and polyphenols and is considered a “superfruit” with various properties including antioxidant, antihyperglycemic and anti-obesity properties (García-Chacón et al., 2023). A recent study found that camu camu extract was able to significantly increase the number of *Akkermansia muciniphila*, *Bifidobacterium* and *Barnesiella* and decrease the abundance of *Lactobacillus*. Meanwhile, mice receiving camu camu extract feces showed the same trend, suggesting that *Akkermansia muciniphila* and *Lactobacillus* may be important drivers of increased energy expenditure and decreased weight gain. In addition, camu camu extract not only increased the levels of chenodeoxycholic acid, deoxycholic acid and ursodeoxycholic acid to activate TGR5, which enhances the response of brown adipocytes to thyroid hormones and activates nonshivering thermogenesis in this tissue, but also reduced IL-1β, IL-6 and LPS levels to prevent gut inflammation (Anhê et al., 2018). In conclusion, camu camu extract has the potential to prevent and treat MAFLD.

*Psidium guajava* L., also known as guava, is an edible fruit that is widely used in folk medicine as an adjunct in the treatment of diabetes (Jiao et al., 2017). It was found that polysaccharides extracted from guava have hypolipidemic effects, and the mechanism of action is highly correlated with its role in regulating the gut microbiota and metabolites (Li Y. et al., 2022). Supplementation with guava polysaccharides increased the relative abundance of *Enterorhabdus* and *Clostridium XlVa*, thereby contributing to the restoration of total SCFA levels and increased levels of acetic acid, propionic acid and butyric acid. The increase in SCFA levels was able to activate hepatic AMPKα and inhibit the expression of peroxisome proliferator-activated receptor γ, thereby suppressing HFD-induced hepatic steatosis and insulin resistance. In addition, guava polysaccharides reduced the relative abundance of *Mucispirillum* to alleviate TNF-α release and NF-κB signaling activation. In conclusion, guava polysaccharides are potential prebiotics with beneficial effects on obesity and MAFLD.

*Morinda citrifolia* L. (noni) is an evergreen tree or shrub of the Rubiaceae family, found primarily in tropical and subtropical regions. In recent years, it has been found that noni fruit polysaccharide not only has physiological activities, such as antitumor, antioxidant and anti-inflammatory activities, but also can play an antilipidemic role by regulating the gut microbiota (Yang X. et al., 2020; Mo et al., 2023). On the one hand, noni fruit polysaccharide increased the relative abundance of *Eubacterium coprostanoligenes*, *Lactobacillus*, *Ruminococcaceae UCG 014*, *Parasutterella*, and *Ruminococcus 1* and decreased the relative abundance of *Prevotella 9*, *Collinsella*, and *Bacteroides*. The altered composition of the gut microbiota increased SCFA production and maintained colonic barrier permeability, thereby reducing serum LPS levels and hepatic lipid accumulation and ultimately ameliorating HFD-induced hepatic oxidative stress and inflammation (Yang X. et al., 2020). On the other hand, noni fruit polysaccharide also increased fecal excretion of fecal-coupled BAs, especially T-α-MCA and T-β-MCA, by modulating the structure of the gut microbiota and activating hepatic and colonic FXR receptors, thus increasing lipid oxidation and energy digestion (Mo et al., 2023). In summary, noni fruit polysaccharide modulates the gut microbiota and its metabolites, which may be its mechanism of action in alleviating MAFLD.

*Polygala japonica* Houtt. is a commonly used herbal medicine that has been shown to possess anti-obesity (Jee et al., 2021) and anti-inflammatory properties (Wang et al., 2007). In addition, Liao et al. (Liao et al., 2023) found that *Polygala japonica Houtt.* may alleviate NASH by regulating gut microbiota composition and amino acid metabolism. On the one hand, *Polygala japonica Houtt.* administration decreased the relative abundance of *Faecalibaculum* and *Lachnospiraceae NK4A136 group* and increased the relative abundance of *Dubosiella*, *Turicibacter* and *Akkermansia*, with the increase in *Akkermansia* modulating the decrease in fecal L-tryptophan, thereby ameliorating the disorders of tryptophan metabolism. In contrast, *Polygala japonica Houtt.* significantly reduced histamine levels as well as fecal and hepatic glutamate accumulation in NASH mice, suggesting that *Polygala japonica Houtt.* not only reduces inflammatory responses by modulating histamine levels but also ameliorates glutamate metabolism disorders. In conclusion, *Polygala japonica Houtt.* treatment significantly altered the composition of the gut microbiota as well as histidine and tryptophan metabolism, which may play a key role in the treatment of NASH.

Quzhou Fructus Aurantii is the dried unripe fruit of *Citrus aurantium* L that is used as a folk medicine and food for treating liver disease; it has hepatoprotective (Lan et al., 2023) and anti-inflammatory effects (Li L. et al., 2020). Recently, Bai et al. (Bai et al., 2019) found that the mechanism of action of Quzhou Fructus Aurantii extract in the treatment of liver disease may be related to its regulation of the gut microbiota. Quzhou Fructus Aurantii extract administration increased the relative abundance of *Akkermansia* and *Alistipes*, which may contribute to the reduction in gut permeability and LPS leakage. On the one hand, Quzhou Fructus Aurantii extract decreased LPS levels and effectively inhibited the TLR4/NF-κB signaling pathway and subsequent proinflammatory cytokine expression. On the other hand, it restored the expression of the gut tight junction proteins Claudin3 and Occludin and improved gut permeability. Furthermore, Quzhou Fructus Aurantii extract reduced the relative abundance of *Dubosiella*, *Faecalibaculum* and *Lactobacillus*. Thus, Quzhou Fructus Aurantii extract ameliorated MAFLD and gut inflammation, at least in part, by altering the gut microbiota.

### 3.3 TCM prescriptions treat MAFLD by regulating the gut microbiota

TCMs, which mainly originated in China, is used to prevent, diagnose and treat diseases or regulate human body functions under the guidance of TCM theories and has been widely used to treat liver diseases, including MAFLD. The treatment of MAFLD by TCM is based on liver protection via multiple mechanisms of action, such as antioxidative stress effects, regulation of lipid metabolism, anti-inflammation effects, anti-fibrosis effects, and regulation of the gut microbiota. Among them, TCM prescriptions have multicomponent and multitarget pharmacological effects, which are compatible with the complex pathogenesis of MAFLD and can alleviate the progression of MAFLD. In recent years, as the gut microbiota has gradually become a new target for the treatment of MAFLD, whether TCM prescriptions exert their therapeutic effects through the gut microbiota has also been gradually investigated. Among them, 12 TCM prescriptions, such as the Salvia-Nelumbinis naturalis formula and Qiang-Gan formula, have been shown to regulate gut microbiota metabolites to improve MAFLD, as shown in **Table 2**.

**Table 2:** Mechanism of action of TCM prescriptions in the treatment of MAFLD by modulating the gut microbiota and its metabolites.

Name	Composition of TCM	Experimental subject	Experimental design	Modulation target (gut microbiota structure)		Modulation target	Ref.
Salvia-Nelumbinis naturalis Formula	*Salvia miltiorrhiza* Bge., *Gynostemma pentaphyllum* (Thunb.) Makino, Polygonum cuspidatum Sieb. et Zucc., *Artemisia capillaris* Thunb., *Nelumbo nucifera* Gaertn	Male C57BL/6 mice	MCD diet supplemented with Salvia-Nelumbinis naturalis Formula extract, 750 mg/kg for 4 weeks	*Lactobacillus*, *Alloprevotella*	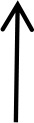	BA metabolism; VDR expression; Gut FXR-FGF15 signaling pathway; TLR4/NF-κB signaling pathway; SIRT1/AMPK signaling pathway	(Li C. et al., 2021; Cao et al., 2022)
Qiang-Gan Formula	*A. capillaris*, *Isatis tinctoria* L., *Angelica sinensis* (Oliv.) Diels., *Paeonia lactiflora* Pall., *S. miltiorrhiza*, *Curcuma wenyujin* Y.H. Chen et C. Ling., *Astragalus membranaceus* (Fisch.) Bge., *Codonopsis pilosula* (Franch.) Nannf., *Alisma orientale* (Sam.) Juz., *Polygonatum kingianum* Coll.et Hemsl., *Rehmannia glutinosa* (Gaertn.) DC., *Dioscorea oppositifolia* L., *Crataegus pinnatifida* Bunge., *Medicated Leaven Massa Medicata Fermentata, Gentiana macrophylla Pall.*, *Glycyrrhiza uralensis* Fisch.	Male C57BL/6 mice	MCD diet supplemented with Qiang-Gan Formula, 400 mg/kg for 4 weeks	*Bacteroides*, *Clostridium*	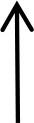	BA metabolism; BSH activity; TGR5 expression; TGR5/NLRP3 inflammatory pathway	(Li Q. et al., 2020)
Zuogui-Jiangtang-Qinggan-Fang	*Rehmannia glutinosa* (Gaetn.) Libosch. ex Fisch. et Mey., *A. membranaceus*, *D. oppositifolia*, *Cormus officinalis* Sieb.et Zucc., *Coptis chinensis* Franch., *S. miltiorrhiza*, *A. capillaris*, *P. cuspidatum*, *C. wenyujin*, *Citrus reticulata* Blanco	Wild-type FVB inbred mice	HFD with Zuogui-Jiangtang-Qinggan-Fang, 7.5 or 15 g/kg for 8 weeks, and ZGJTQG was administered from the ninth week	Muribaculaceae, Lactobacillaceae, *Akkermansia* Lachnospiraceae, Desulfovibrionaceae	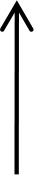 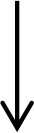	SCFA production; Gut barrier; Insulin resistance	(Zou et al., 2023; Zou et al., 2022)
Yinchen Linggui Zhugan Decoction	*A. capillaris*, *Gardenia jasminoides* Ellis, *Rheum palmatum* L., *Poria cocos* (Schw.) Wolf, *Cinnamomum cassia* (L.) C. Presl., *Atractylodes macrocephala* Koidz., *G. uralensis*	Male Sprague–Dawley rats	HFD with Yinchen Linggui Zhugan Decoction, 19.2 g/kg/day for 4 consecutive weeks, and YLZD was administered from the eleventh week	SCFA-producing bacteria Christensenellaceae, Muribaculateae, Prevotellaceae	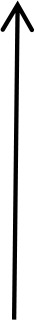	SCFA production; Oxidative stress; SIRT1/Nrf2 signaling pathway	(Jiang et al., 2022b)
Zhishi Daozhi Decoction	*Citrus aurantium* L., *R. palmatum* L., *C. chinensis*, *Scutellaria baicalensis* Georgi, *Massa Medicata Fermentata*, *A. macrocephala*, *P. cocos*, *A. orientale*	Male C57BL/6 mice	HFD with Zhishi Daozhi Decoction, 14.5 g/kg/d for 4 consecutive weeks, and Zhishi Daozhi Decoction was administered from the eleventh week	*Faecalibacterium*, *Bacteroidetes* *Brautia*, *Colidextribacter*	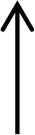 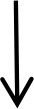	SCFA production; Gut barrier; Gut inflammation	(Bi et al., 2022)
Shenling Baizhu powder	*Panax ginseng* C.A.Mey., *P. cocos*, *A. macrocephala*, *D. oppositifolia*, *Dolichos lablab* L., *N. nucifera*, *G. uralensis*, *Coix lacryma-jobi* L.var.*ma-yuen* (Roman.) Stapf, *Platycodon grandiflorum*(Jacq.)A.DC., *Amomum villosum* Lour.	Male Sprague–Dawley rats	Fed a HFD and intragastrically administered 30 g/kg/day Shenling Baizhu powder for 16 weeks	SCFA-producing bacteria; *Bifidobacterium*, *Anaerostipes*	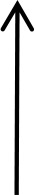	LPS production; LPS/TLR4/NLRP3 inflammatory signaling pathway; Gut permeability and gut mucosa; Lipid metabolism	(Zhang et al., 2018; Pan et al., 2021; Tang et al., 2020)
Hongqijiangzhi Fang	*A. membranaceus*, *Semen oryzae cum monasco*, *A. capillaris*, *Lycium barbarum* L., *Curcuma Longa* L., *N. nucifera*, *Magnolia officinalis* Rehd.et Wils	Specific pathogen-free male Sprague–Dawley rats	HFD with Hongqijiangzhi Fang, 19.05 g/kg/day for 16 weeks	Enterobacteriaceae *F. rappini*	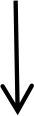	LPS production; NLRP3 inflammatory pathway; Gut inflammation	(Liang et al., 2018)
Qushi Huayu Decoction	*A. capillaris*, *G. jasminoides*, *Fallopia japonica*, *C. longa*, *Hypericum japonicum* Thunb. ex Murray	Male C57BL/6 mice	HFD with Qushi Huayu Decoction, 9.3 g/kg/d for 4 consecutive weeks, and Qushi Huayu Decoction was administered from the thirteenth week	SCFA-producing bacteria *Butyricimonas*, *Eubacterium*, *Collinsella*; *Escherichia*/*Shigella*	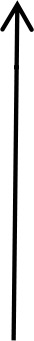	Gut epithelial barrier; LPS production; Gut inflammation	(Leng et al., 2020; Feng et al., 2017)
Huazhi-Rougan Formula	*A. capillaris*, *Cassia obtusifolia* L., *R. palmatum*, *A. orientale*, *Polyporus umbellatus* (Pers.) Fries., *C. pinnatifida*, *Atractylodes lancea* (Thunb.) DC., *A. macrocephala*, *C. reticulata*, *Trichosanthes kirilowii* Maxim., *Ligustrum lucidum* Ait., *Eclipta prostrate* L., *L. barbarum*, *Cirsium setosum* (Willd.) Besser, *Bupleurum chinense* DC., *G. uralensis*	Male C57BL/6J mice	MCD with Huazhi-Rougan Formula, 3 or 6 g/kg/d for 4 weeks	Lactobacillaceae, Bifidobacteriaceae, Clostruduaceae	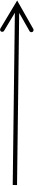	Gut barrier; BA metabolism; BA-transporting portal transport molecules (MRP2/3, OSTα/β)	(Li C. et al., 2022)
Er-Chen Decoction	*Pinellia ernate* (Thunb.) Breit., *C. aurantium*, *Smilax glabra Roxb.*, *G. uralensis*	Specific pathogen-free-grade male Sprague–Dawley rats	HFD with Er-Chen Decoction, 4.5 or 9 g/kg/d for 12 weeks	*Lactobacillus*, *Dubosiella*, *Akkermansia*, *Intestinimonas* *Alloprevotella, C.saccharimonas*	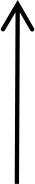 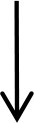	Vitamin B6 metabolism; Taurine and hypotaurine metabolism, cysteine and methionine metabolism;LPS translocation; Oxidative stress	(Liu H. et al., 2021; Miao et al., 2022)
Sheng-Jiang Powder	*Bombyx batryticatus*, *Cicadae periostracum*, *C. Longa*, *R. palmatum*	Male C57BL/6 mice	HFD was fed for 12 weeks, and Sheng-Jiang Powder was administered from the seventh week for 6 weeks	SCFA-producing bacteria Erysipelotrichaceae *Roseburia* *Desulfovibrio*	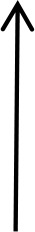 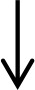	SCFA production; LPS production; Akt/mTOR/S6 signaling pathway; Gut inflammation	(Li J. et al., 2020)
Sanwei Ganjiang Powder	*Zingiber officinale* Rosc., *Amomum kravanh* Pierre ex Gagnep., *Myristica fragrans* Houtt.	Specific pathogen-free inbred BALB/c mice (half male and half female)	Subcutaneous injection of 40% CCl_4_ oil solution (5 mL/kg was first injected, followed by 3 mL/kg) twice a week for 8 weeks, and Sanwei Ganjiang Powder, 0.165 or 0.66 g/kg was given from the third week	Promoted beneficial bacteria and inhibited harmful bacteria, reduced the Firmicutes/Bacteroidetes ratio		BA reabsorption and excretion; Gut barrier; Gut inflammation	(Li N. et al., 2019)

MAFLD, Metabolic-associated fatty liver disease; TCM, traditional Chinese medicine; HFD, high-fat diet; MCD, methionine/choline-deficient diet; BA, bile acid; SCFA, short-chain fatty acid; LPS, lipopolysaccharide; BSH, bile salt hydrolase; FXR, farnesoid X receptor; TLR4, Toll-like receptor-4; TGR5, Takeda G-protein-coupled receptor 5; MRP2/3, multidrug resistance‐associated protein 2/3; OSTα/β, organic solute transporter alpha/beta; NF-κB, nuclear factor kappa-B; Nrf2, nuclear factor erythroid 2-related factor 2; NLRP3, nucleotide-binding oligomerization domain, leucine-rich repeat and pyrin domain-containing 3; SIRT1, silent information regulator 1; AMPK, adenosine 5’-monophosphate (AMP)-activated protein kinase; FGF15, recombinant fibroblast growth factor 15; Akt, serine/threonine kinase Akt; mTOR, mammalian target of rapamycin.

"↑" indicates an increase in the relative abundance of bacteria; "↓" indicates a decrease in the relative abundance of bacteria.

The Salvia-Nelumbinis naturalis formula, initially called a lipid-lowering granule, is an herbal compound designed based on TCM theories and has been used clinically for the treatment of MAFLD and alleviation of liver steatosis with significant beneficial effects and few side effects. The alleviation of MAFLD by Salvia-Nelumbinis naturalis may be achieved by regulation of the gut microbiota and BAs (Li C. et al., 2021; Cao et al., 2022). Salvia-Nelumbinis naturalis supplementation restored the relative abundances of the beneficial bacteria *Lactobacillus* and *Alloprevotella*, increased (3α,5β,12α)-3,12-dihydroxy-24-norcholan-23-oic acid levels, and activated the FXR signaling pathway, which helped counteract LPS-induced impairment of the gut epithelial barrier and ameliorated metabolic disorders and liver disease (Li C. et al., 2021). In addition, Salvia-Nelumbinis naturalis counteracts the progression of NASH by increasing the proportion of secondary BAs and activating the endogenous vitamin D receptor (Cao et al., 2022).

The Qianggan formula is a traditional formula used in China for the treatment of liver diseases. Studies have shown that Qianggan formula treatment reverses gut microbiota disturbance and elevates the relative abundances of *Bacteroides* and *Clostridium*, which are involved in the BSH uncoupling process and therefore lead to an increase in secondary bile acid lithocholic acid (LCA) levels. In addition, the expression of TGR5 was increased in the livers of QGE-treated mice with NASH, which could be attributed to the increased LCA activation by TGR5, which ameliorated metabolic disorders and blocked nucleotide-binding oligomerization domain, leucine-rich repeat and pyrin domain-containing 3 (NLRP3) inflammasome-dependent inflammation in mice (Li Q. et al., 2020). Thus, the therapeutic effect of the Qianggan formula for MAFLD may be related to the interaction between the gut microbiota and BA metabolism.

Zuogui-Jiangtang-Qinggan-Fang, a new herbal formulation, has been clinically used to treat T2DM and fatty liver disease. Zuogui-Jiangtang-Qinggan-Fang reportedly elevated the relative abundances of Muribaculaceae and Lactobacillaceae and reduced the relative abundances of Lachnospiraceae and Desulfovibrionaceae (Zou et al., 2023). Furthermore, Zuogui-Jiangtang-Qinggan-Fang promoted the growth of the SCFA-producing bacterium *Akkermansia*, which repaired the gut epithelial barrier and alleviated insulin resistance by increasing the concentration of SCFAs (Zou et al., 2022). Thus, Zuogui-Jiangtang-Qinggan-Fang treatment may prevent and alleviate MAFLD by regulating the gut microbiota composition.

Yinchen Linggui Zhugan decoction is a classic combination of two well-known herbal prescriptions, namely, Linggui Zhugan and Yinchenhao decoctions, and has been used to treat hepatobiliary diseases and metabolic syndrome (Jiang et al., 2022a). Yinchen Linggui Zhugan decoction can enrich the SCFA-producing bacteria Christensenellaceae, Muribaculateae and Prevotellaceae to increase the gut SCFA content, especially Christensenellaceae, which is closely correlated with acetic acid, butyric acid and total SCFAs. The increase in butyric acid not only provides energy to gut epithelial cells but also activates the silent information regulator 1/Nrf2 signaling pathway and increases the expression of downstream antioxidant factors, which in turn ameliorates oxidative stress (Jiang et al., 2022b). These results suggest that Yinchen Linggui Zhugan decoction can alleviate MAFLD by modulating the gut microbiota and butyric acid levels.

Zhishi Daozhi decoction, a water decoction of herbs prescribed for use in Zhishi Daozhi pills, has been shown to have hepatoprotective and lipid-lowering effects. The effect of the Zhishi Daozhi decoction in the treatment of MAFLD may be achieved by modulating the gut microbiota (Bi et al., 2022). Zhishi Daozhi decoction may restore gut microbiota imbalance by promoting the growth of *Faecalibacterium* and *Bacteroidetes* and inhibiting the growth of *Brautia* and *Colidex*. The reconstituted gut microbiota also increases the amount of SCFAs in the gut, which not only reduces energy and fat deposition in the body but also provides energy to gut epithelial cells and ensures adequate expression of gut transepithelial resistance and tight junction proteins, thus improving the gut barrier and preventing gut inflammation.

Shenling Baizhu powder is a classic herb with a history of clinical use for thousands of years. Shenling Baizhu powder may alleviate MAFLD by regulating the gut microbiota. PSP not only increased the abundances of *Bifidobacterium* and *Anaerostipes* but also promoted the growth of SCFA-producing bacteria, accelerating the production of SCFAs that maintain normal gut permeability and protect the gut mucosa (Zhang et al., 2018). On the other hand, Shenling Baizhu powder can reduce LPS levels and inhibit TLR4 expression to suppress NLRP3 inflammatory vesicle activation and interleukin-1β (IL-1β) release (Pan et al., 2021). In addition, Shenling Baizhu powder can increase lipocalin levels in the liver and serum and inhibit SREBP-1c expression, thereby regulating systemic lipid metabolism and reducing hepatic lipid accumulation (Tang et al., 2020).

Hongqijiangzhi Fang is a spleen-strengthening herbal formula. Studies have shown that Hongqijiangzhi Fang can alleviate lipid metabolism disorders and reduce liver fat deposition, suggesting that it has a therapeutic effect on MAFLD. Liang et al. (Liang et al., 2018) reported that Hongqijiangzhi Fang may enhance gut barrier integrity by reducing the relative abundances of Enterobacteriaceae and *F. rapa*, thereby reducing LPS levels and suppressing gut inflammation. In addition, Hongqijiangzhi Fang may inhibit hepatic steatosis by reducing Enterobacteriaceae translocation and inhibiting NLRP3 inflammasome activation.

Qushi Huayu decoction is derived from Yinchenhao decoction, a classic prescription dating back to the Han Dynasty documented in the book Treatise on Cold Damage Disorders, which is one of the popular formulas for the treatment of MAFLD in China. In recent years, it has been shown that Qushi Huayu decoction can elevate the relative abundances of SCFA-producing bacteria *Butyricimonas* and *Eubacterium* to increase the SCFA content, which can provide energy to local gut epithelial cells and help maintain the gut epithelial barrier (Leng et al., 2020). Feng et al. (Feng et al., 2017) also reported that Qushi Huayu decoction treatment suppressed the HFD-induced increase in *Escherichia/Shigella* and elevated the relative abundance of the SCFA-producing bacteria *Collinsella*. In addition, Qushi Huayu decoction was able to reduce the serum LPS concentration, which may be associated with an improvement in gut inflammation. Therefore, Qushi Huayu decoction may alleviate MAFLD by repairing the gut barrier and modulating the gut microbiota composition.

The Huazhi-Rougan formula contains flavonoids, alkaloids and lactones as the main components and has been widely used to treat MAFLD and its complications. Studies have shown that Huazhi-Rougan formula treatment counteracts MCD-induced hepatic steatosis and inflammation by regulating gut ecological dysregulation and promoting fecal BA excretion (Li C. et al., 2022). The Huazhi-Rougan formula decreased the enrichment of HFD-associated bacteria and elevated the enrichment of Lactobacillaceae, Bifidobacteriaceae, and Clostridiaceae, which are involved in the uncoupling, oxidation and isomerization of BAs and contribute to the formation of secondary BAs. In addition, the Huazhi-Rougan formula alleviated gut barrier damage and alleviated MAFLD by inhibiting the expression of the intestinal BA transporter and the BA-transporting portal transport molecules multidrug resistance‐associated protein 2/3 and organic solute transporter alpha/beta, promoting fecal BA excretion and reducing secondary BA accumulation in the gut.

Er-Chen decoction, a common TCM formula originating from the Taiping Huimin Formula Bureau, is a basic herbal formula for treating dry dampness and phlegm and has been used to treat various phlegm-dampness diseases, such as MAFLD. Many studies have investigated the mechanism by which Er-Chen decoction improves lipid metabolism disorders, and Liu et al. (Liu H. et al., 2021) reported that Er-Chen decoction could reduce LPS translocation and reduce liver inflammation by reversing HFD-induced gut barrier dysfunction through regulation of the gut microbiota. Miao et al. (Miao et al., 2022) showed that Er-Chen decoction could alleviate MAFLD by inhibiting oxidative stress, reducing inflammatory responses, ameliorating gut microbiota dysbiosis, and increasing pyridoxal (vitamin B6) levels. The increase in pyridoxal concentrations not only inhibited lipid synthesis but also reduced oxidative and inflammatory stress. Thus, the effect of Er-Chen decoction on MAFLD may be achieved through the gut-liver axis pathway involving the gut microbiota.

Sheng-Jiang powder, derived from the “wan bing hui chun” compiled by Ting-Xian Gong of the Ming Dynasty, is a typical herbal formula for restoring the “ascending and descending abnormalities” of the spleen qi and has been used to treat MAFLD. Sheng-Jiang powder supplementation increased the relative abundances of the SCFA-producing bacteria Erysipelotrichaceae and *Roseburia* and decreased the relative abundance of the harmful bacteria *Desulfovibrio* (Li J. et al., 2020). These findings suggest that Sheng-Jiang powder may alleviate gut leakage by increasing SCFA levels to ameliorate gut barrier damage and may also ameliorate gut inflammation by inhibiting LPS production or entry into the gut. Thus, the protective effect of Sheng-Jiang powder against HFD-induced MAFLD may be partially attributed to its regulation of the gut microbiota.

Sanwei Ganjiang powder, also known as Jia Ga Song Tang, not only has antipyretic and antidiarrheal effects and promotes gas circulation but also has anti-inflammatory effects, protects the gastrointestinal mucosa, and enhances antioxidant and hepatoprotective pharmacological effects. Li et al. (Li N. et al., 2019) reported that Sanwei Ganjiang powder could alleviate liver injury by regulating the gut microbiota and restoring BA homeostasis. Sanwei Ganjiang powder may regulate the Firmicutes/Bacteroidetes ratio, promote beneficial bacteria and inhibit harmful bacteria. Its modifying effects on the gut microbiota lead to upregulation of the expression of specific BA metabolic enzymes, as well as reabsorption and efflux transporters, thus promoting the detoxification, reabsorption and excretion of TBAs and reversing disrupted BA homeostasis. In addition, Sanwei Ganjiang powder increased the expression of gut tight junction proteins such as ZO-1 and Occludin and repaired the gut barrier to inhibit LPS-induced gut inflammation. Therefore, treatment with Sanwei Ganjiang powder may be a potential strategy for treating MAFLD by maintaining the gut-liver axis.

## 4 Perspective

MAFLD has become the most common chronic liver disease in the world, and its incidence is increasing annually (Lin et al., 2021). However, due to the complexity of the pathogenesis of MAFLD, pathogenic processes such as insulin resistance, lipotoxicity, oxidative stress, altered immune or cytokine or mitochondrial function, and apoptosis, are collectively involved in the development and progression of MAFLD. At the same time, the pathogenetic process of MAFLD is often accompanied by a variety of complications; therefore, drug therapy needs to simultaneously account for the prevention and treatment of hepatic steatosis, inflammation, hepatocellular injury and fibrosis, as well as the treatment of complications, which is very difficult. Second, NASH requires a liver biopsy to determine if the patient has NASH and what stage of liver fibrosis the patient is currently in. However, liver biopsies are invasive and not easily accepted by patients. In clinical practice, it is not very feasible to repeat liver biopsies to assess whether liver injury and liver fibrosis have improved. While it is feasible that current research on NASH drug therapy focuses on improvements in MAFLD activity scores, the scores are subjective. Therefore, there is an urgent need to develop and validate a set of noninvasive methods for assessing NASH fibrosis and documenting its progression or reversal to help enhance accelerated new drug development for MAFLD and NASH. For these and other reasons, there are currently no FDA-approved therapeutic drugs. Therefore, it is essential to find safe and effective anti-MAFLD drugs. In recent years, the relationship between the gut microbiota and metabolic diseases has been widely recognized (Boulangé et al., 2016). An imbalance in the gut microbiome exacerbates disease progression, and targeted modulation of the gut microbiome has emerged as a viable strategy for the prevention and treatment of MAFLD. Plant natural products involve multiple pathways, multiple targets and few side effects. Plant natural products exert their effects in the prevention and treatment of MAFLD not only by regulating lipogenic pathways, downregulating inflammatory pathways, improving insulin sensitivity, and ameliorating oxidative stress but also by regulating the gut microbiota and its metabolites, for example, via the gut-liver axis (Wang L. et al., 2023).

Modern pharmacokinetic studies have shown that most plant natural products are difficult to absorb or have poor absorption rates. Berberine, for example, is less than 1% orally available, but it has been shown to have therapeutic activity against a variety of diseases. This may be because berberine can be converted to dihydroberberine by enzymes produced by the gut microbiota, which increases its absorption and subsequently utilization. In addition, some “zero absorption” natural ingredients whose main function may be to provide nutrients for the gut microbiota, improve the activity of the gut microbiota and exert their effects through the release of active molecules by the gut microbiota. The contribution and function of the gut microbiota through biotransformation of the components of plant natural products or through enhancement of the activity of the gut microbiota cannot be ignored. Therefore, targeting the gut microbiota for the treatment of MAFLD may be more conducive to ensuring the efficacy of plant natural products in controlling the development of this disease.

Recent research on the relationships among the gut microbiota, gut permeability and metabolic disorders has provided new perspectives on the treatment of MAFLD. In the clinical application of plant natural products for MAFLD treatment, many patients are treated based on the principle of “strengthening the spleen” from the perspective of “spleen deficiency”, which is often accompanied by the improvement of gut symptoms such as diarrhea and defecation. Numerous studies have shown that plant natural products can support the growth of beneficial bacteria in the gut, inhibit the growth of harmful bacteria, maintain the balance of beneficial and harmful bacteria, and improve gut permeability. The study of “gut-liver” axis interactions provides a reference and method for the treatment of MAFLD by plant natural products and the gut microbiota, which allows exploration of the pharmacological mechanism of plant natural products in the treatment of MAFLD from the perspective of gut microenvironment and gut barrier regulation.

Although the gut microbiome is a hot topic and a new entry point for studying the mechanism of action of plant natural products, the mechanism cannot be simply attributed to alterations in the gut microbiome. Alterations in the gut microbiota may be one result of plant natural product interventions in the organism, but not all the pathways involved in these effects are known, and additional experimental evidence needs to be obtained. Based on the above, in addition to using 16S ribosomal DNA identification to determine the changes in the gut microbiota, we need to further explore the role of the gut microbiota in the efficacy of plant natural products through FMT, testing with germ-free mice, antibiotic intervention and other methods.

In addition, the changes in the gut microbiota are unstable. The gut microbiota is easily affected by the external environment. Although the self-repair ability of the gut microbiota can restore its balance after short-term disturbance, long-term disturbance often causes gut microbiota disorders; that is, differences in experimental environments and conditions may lead to changes in the gut microbiota. Therefore, it is particularly important and more convincing to identify stable and changing bacterial species, such as pathogenic bacteria or beneficial bacteria, through the instability of the gut microbiota. Crossomics data need to be integrated and analyzed with data from additional high-throughput omics technologies, such as metagenomics, metabolomics, and bioinformatics. From the perspective of the composition and function of the gut microbiota and the interaction between the gut microbiota and host metabolism, key strains and their related metabolites will need to be explored in depth.

Numerous plant natural compounds, such as berberine, resveratrol, curcumin, and puerarin, have been found to alleviate MAFLD. However, TCM prescriptions often achieve better therapeutic effects and have the characteristics and advantages associated with the holistic use of Chinese medicine in the treatment of complex diseases. TCM prescriptions are safe and effective modern Chinese medicines with a clear mechanism of action formed on the basis of TCM enhanced by modernization and pharmacological research and represent one of the future development directions of Chinese medicine. However, there are some limitations in the current development of plant natural products for the treatment of MAFLD. One of the limitations is that the mechanisms of action of plant natural products are complex, and it is difficult to clarify the specific mechanisms of action. Second, the limitation of plant natural products emphasizes the influence of the place of origin on their quality and efficacy. Authentic herbs not only have unique environmental conditions in terms of their origin but also are subjected to specific natural factors and artificial management during their growth process, which makes them of superior quality. In conclusion, the interaction between plant natural products and the gut microbiota provides a safe, effective, and feasible therapeutic approach and modality for treating MAFLD.

## 5 Conclusion

This narrative review systematically summarizes the effects of plant natural products on the prevention of MAFLD by regulating the gut microbiota and its metabolite-related pathways, which provides feasible ideas for further exploring safer and more effective plant natural products as drugs for the prevention and treatment of MAFLD. Moreover, on the basis of this review, it is necessary to strengthen the study of the interactions among plant natural products, the gut microbiota and the human body. In the future, more attention should be given to the role of the gut microbiota in the pharmacodynamic effect of Chinese medicine and the contribution of the effects of the gut microbiota on host metabolism to the development of disease or pharmacodynamic effects.

## Author contributions

TC: Writing – original draft, Writing – review & editing. XS: Writing – original draft, Writing – review & editing. XX: Software, Visualization, Writing – review & editing. LD: Software, Visualization, Writing – review & editing. SL: Software, Visualization, Writing – review & editing. MX: Software, Visualization, Writing – review & editing. YH: Software, Visualization, Writing – review & editing. LZ: Investigation, Resources, Writing – review & editing. TL: Investigation, Resources, Writing – review & editing. XW: Investigation, Resources, Writing – review & editing. YF: Investigation, Resources, Writing – review & editing. ZX: Conceptualization, Writing – review & editing. CW: Conceptualization, Writing – review & editing. MW: Conceptualization, Writing – review & editing. JL: Conceptualization, Writing – review & editing. YZ: Project administration, Supervision, Writing – review & editing. WS: Conceptualization, Investigation, Project administration, Supervision, Writing – review & editing. LL: Project administration, Supervision, Writing – review & editing.

## Glossary

**Table:**

MAFLD	metabolic-associated fatty liver disease
NAFLD	nonalcoholic fatty liver disease
T2DM	type 2 diabetes mellitus
TCM	traditional Chinese medicine
NAFL	simple fatty liver
NASH	steatohepatitis
HCC	hepatocellular carcinoma
FDA	Food and Drug Administration
BA	bile acid
LPS	lipopolysaccharide
SCFA	short-chain fatty acid
TMAO	trimethylamine-N-oxide
FXR	farnesoid X receptor
VDR	vitamin D receptor
PXR	pregnane X receptor
TGR5	Takeda G protein-coupled receptor 5
BSH	bile salt hydrolase
TBA	total bile acid
SHP	small heterodimer partner
SREBP-1c	sterol regulatory element-binding protein 1c
PPAR-α	peroxisome proliferator-activated receptor alpha
CYP7A1	cytochrome P450 7A1
AgRP	Agouti-related protein
NPY	neuropeptide Y
GPR41/GPR43	mammalian G protein-coupled receptors 41/43
GPR109A	G protein-coupled receptor 109 A
AMPK	adenosine 5’-monophosphate (AMP)-activated protein kinase
TLR4	Toll-like receptor 4
TMA	trimethylamine
VD	vitamin D
NF-κB	nuclear factor kappa-B
HFD	high-fat diet
TNF-α	tumor necrosis factor alpha
Nrf2	nuclear factor erythroid 2-related factor 2
LXR-α	liver X receptor alpha
ZO-1	zonula occludens protein 1
CYP27A1	cytochrome P450 27A1
BSEP	bile salt export pump
NTCP	Na^+^-taurocholate cotransporting polypeptide
FGF15	recombinant fibroblast growth factor 15
T-β-MCA	Tauro-β-muricholic acid
T-α-MCA	Tauro-α-muricholic acid
MCD	methionine–choline-deficient
FMT	fecal microbiota transplantation
TCDCA	taurochenodeoxycholic acid
TUDCA	tauroursodeoxycholic acid
LCA	lithocholic acid
NLRP3	nucleotide-binding oligomerization domain, leucine-rich repeat and pyrin domain-containing 3
IL-1β	interleukin-1β
